# Supplementary material for: Voices from the margins: How national stories are linked with support for populist radical right parties
Source: PLoS One. 2024 Aug 12;19(8):e0305554. doi: 10.1371/journal.pone.0305554 (PMC11318891; doi:10.1371/journal.pone.0305554)
Supplement: S1 Appendix — (DOCX) [file pone.0305554.s001.docx]

**Voices from the margins: How national stories are linked with support for populist radical right parties**

Supporting Information

**Online Appendix A: survey companies, sampling techniques, and descriptive statistics of the samples**

The four surveys were administered online in the respondents’ native language. The Danish survey was conducted in June 2015, during the parliamentary election, on a sample of 1,010 respondents; the Dutch survey (N = 1,448) – during the 2012 parliamentary election; the British survey (N = 1,002) – during the 2015 parliamentary election; and the American survey (N = 1,001) – during the 2016 presidential election. All survey companies (ICM Unlimited) compensate participants according to local standards for opt-in internet panels. Survey respondents were all adults 18 and over, provided informed consent in the beginning of the survey, and all data is anonymous with no identifying features.

The Danish, British and American surveys were administered by ICM Unlimited, on a sample matched to the general population by age, gender, ethnicity and region. Tables A1, A2 and A3 report summary statistics for the Danish, British and American samples.

**Table A1. Sample profile - Denmark**

Representative quotas were set on age, gender, ethnicity and region. The table below provides a sample profile, detailing weighted and un-weighted figures.

|  | **Target number of respondents** | **Target % of respondents** | **Achieved number of respondents** | **Achieved % of respondents** |
| --- | --- | --- | --- | --- |
| **Gender** |  | | | |
| Male | 500 | 50.0 | 489 | 48.9 |
| Female | 500 | 50.0 | 521 | 52.1 |
| **Age** |  | | | |
| 18-24 | 150 | 15.0 | 91 | 9.0 |
| 25-34 | 140 | 14.0 | 105 | 10.4 |
| 35-44 | 170 | 17.0 | 187 | 18.5 |
| 45-54 | 170 | 17.0 | 250 | 24.8 |
| 55+ | 380 | 38.0 | 377 | 37.3 |
| **Region** |  | | | |
| Hovedstaden | 310 | 31.0 | 293 | 29.0 |
| Midtjylland | 230 | 23.0 | 218 | 21.6 |
| Nordjylland | 100 | 10.0 | 97 | 9.6 |
| Sjaelland | 150 | 15.0 | 168 | 16.6 |
| Stddanmark | 210 | 21.0 | 234 | 23.2 |

**Table A2. Sample profile – UK**

The table below provides a sample profile, detailing weighted and un-weighted figures. The sample was set at n=1,000.

|  | **Target number of respondents** | **Target % of respondents** | **Achieved number of respondents** | **Achieved % of respondents** |
| --- | --- | --- | --- | --- |
| **Gender** |  | | | |
| Male | 486 | 48.6 | 486 | 48.5 |
| Female | 514 | 51.4 | 516 | 51.5 |
| **Age** |  | | | |
| 18-24 | 117 | 11.7 | 117 | 11.7 |
| 25-34 | 172 | 17.2 | 165 | 16.5 |
| 35-44 | 168 | 16.8 | 170 | 17.0 |
| 45-54 | 179 | 17.9 | 181 | 18.1 |
| 55-64 | 144 | 14.4 | 144 | 14.4 |
| 65+ | 220 | 22.0 | 225 | 22.5 |
| **Region** |  | | | |
| Scotland | 89 | 8.5 | 90 | 9.0 |
| North East | 43 | 4.1 | 43 | 4.3 |
| North West | 111 | 11.1 | 111 | 11.1 |
| Yorkshire and the Humber | 89 | 8.3 | 88 | 8.8 |
| East Midlands | 72 | 7.2 | 71 | 7.1 |
| West Midlands | 88 | 8.8 | 87 | 8.7 |
| Wales | 52 | 4.9 | 52 | 5.2 |
| East of England | 93 | 9.3 | 86 | 8.6 |
| London | 135 | 12.9 | 135 | 13.5 |
| South East | 137 | 13.7 | 135 | 13.5 |
| South West | 89 | 8.5 | 89 | 8.9 |
| Northern Ireland | 28 | 2.8 | 15 | 1.5 |

**Table A3. Sample profile - USA**

Representative quotas were set on age, gender, and region. The table below provides a sample profile, detailing weighted and un-weighted figures.

|  | **Target number of respondents** | **Target % of respondents** | **Achieved number of respondents** | **Achieved % of respondents** |
| --- | --- | --- | --- | --- |
| **Gender** |  | | | |
| Male | 500 | 50.0 | 490 | 49.0 |
| Female | 500 | 50.0 | 511 | 51.0 |
| **Age** |  | | | |
| 18-24 | 170 | 17.0 | 162 | 16.2 |
| 25-34 | 170 | 17.0 | 181 | 18.1 |
| 35-44 | 160 | 16.0 | 165 | 16.5 |
| 45-54 | 170 | 17.0 | 169 | 16.9 |
| 55+ | 330 | 33.0 | 324 | 32.4 |
| **Region** |  | | | |
| Northeast | 180 | 18.0 | 187 | 18.7 |
| Midwest | 220 | 22.0 | 211 | 21.1 |
| South | 370 | 37.0 | 343 | 34.3 |
| West | 240 | 24.0 | 260 | 26.0 |

**Sample profile – The Netherlands**

In the Netherlands data was collected by IntomartGfk, a commercial market research company. The polling company GfK conducted the survey in alignment with ESOMAR and ISO 26362 guidelines for representative computer-assisted web interviewing panels. The respondents represent a stratified sample of a large multi access panel (MAP) of respondents (N above 100,000). Multiple recruitment strategies were used to enable a large-scale sample that match the population characteristics of the Dutch population (95% internet penetration), including a not-so-heavy internet use on the average.

Approximately 31% of MAP respondents were recruited from various difficult to reach population subgroups. Stratification was based on turnout and voting choice at the previous election, internet use, age, education, gender and Nielsen-regions. The stratification guaranteed that difficult to reach groups such as nonvoters, new voters, and voters for parties with low-education voters proportionally represented. The number of respondents amounted to 1,448.

**Online Appendix B: Categorization of Stories**

**Intercoder Reliability**

Story components, i.e., past and future events/issues, for all respondents in each country were compiled from the data (*n*=680) and classified by human coders into three story types: *Survival, Self-expression* and *Us-versus-Them*. Two of the PI's discussed and coded together a subset of the data (80 story components). Two expert human coders read the story components and assigned them to story types (one story component could be assigned to one or more types, or to none). For example, the future story *Exit the EU* was assigned to *Us versus Them* type. The past component *Churchill's era* (cited by 1.4% of respondents in Britain) wasn’t assigned to any of the three story types, while *World War II* was assigned to both the *Survival* and the *Us versus Them* story types. The two coders discussed cases of disagreement to better define what should be included or not included in each story type. Intercoder reliability for the three story-types, calculated on another subset of the data (120 story components), showed high values: between .81 for *Us versus Them* and .96 for *Survival*.

**Examples of Story Component for Each Story Type**

*Survival*. In the U.K., past events: WWI, WWII, the Falkland War, Napoleonic Wars, Roman occupation, the Industrial Revolution, and the introduction of the welfare state; future concerns, a strong economy, job opportunities, and dealing with terrorism. In the U.S., past events: the September 11 attacks, the Civil War, the 2003 invasion of Iraq, and terror: future concerns: gun control, more jobs, end of terror, and end of poverty. In Denmark, past events: WWII, losing Southern Jutland, the introduction of the welfare state, and the conquest of England; future concerns: employment opportunities, a strong economy, tax reform, and dealing with the Muslim problem. In the Netherlands, past events: the Eighty Years’ War, WWII, VOC era, industrialization, the formation of trade unions, and economic crisis; future concerns: economic growth, pensions, and prosperity.

*Self-expression*. In the UK, past developments: signing the Magna Carta, women's rights, and secularization of Britain; future concerns: peace, equality, better quality of life, and environmental policy. In the U.S., past issues: freedom, rights, equality, democracy, and diversity; future concerns: social harmony, end of racism, and peace. In Denmark, past developments: women's rights, the introduction of democracy, and the abolition of serfdom; future issues: environmental policy, integration, and tolerance. In the Netherlands, past developments: suffrage, freedom, democracy, and secularization; future issues: a more tolerant society, environmental policy, peace and civil rights.

*Us versus Them*. In the UK, past events: WW2, WW1, Napoleonic Wars, Falkland War, Enoch Powell's warning speech, and immigration policy; future concerns: immigration, exiting the EU, reducing the power of the EU, and dealing with terrorism. In the US, past events: the September 11 attacks, immigration, the Revolutionary War, the 2003 invasion of Iraq, and the deployment of atomic bombs on Japan; future concerns: immigration and end of terror. In Denmark, past events: losing Southern Jutland, EU referendums, WWI and WWII: future concerns: immigration, exiting the EU, dealing with the Muslim problem, and reducing the power of the EU. In the Netherlands, past events: the Eighty Years’ War, WWII, immigration, 9/11, and the Cold War; future concerns: exiting the EU and limiting or stopping immigration.

**Examples of Disagreement Between Coders and “Grey” Areas**

- The two expert coders disagreed on the classification of several story components. One such disagreement was over healthcare, or the introduction of National Health Service: one coder assigned it to the *Survival* story type, while the other – to *Self-expression*. As healthcare is germane to both quality of life (self-expression) and physical survival, this disagreement stands to reason. Ultimately, we decided not to assign this component to any story type.
- Several story components pertained to populist party voters. Dwelling on such issues as PVV (the Dutch populist party) or to Geert Wilders (PVV’s leader) implies that the individual is likely to vote for a populist party. However, as these components did not relate to any of the three story-types, we did not classify them.

*Table B1*. Frequency of Story Types in Denmark.

|  | Past | Future |
| --- | --- | --- |
| Survival | 243/986 (25%) | 280/1002 (28%) |
| Self-expression | 110/986 (11%) | 182/1002 (18%) |
| Us versus Them | 241/986 (24%) | 164/1002 (16%) |

*Table B2*. Frequency of Story Types in the Netherlands.

|  | Past | Future |
| --- | --- | --- |
| Survival Values | 561/1448 (39%) | 346/1448 (24%) |
| Self-expression Values | 125/1448 (9%) | 396/1448 (27%) |
| Us versus Them | 385/1448 (27%) | 100/1448 (7%) |

*Table B3*. Frequency of Story Types in the U.K.

|  | Past | Future |
| --- | --- | --- |
| Survival Values | 453/996 (45%) | 142/998 (14%) |
| Self-expression Values | 96/996 (10%) | 143/998 (14%) |
| Us versus Them | 395/996 (40%) | 196/998 (20%) |

*Table B4*. Frequency of Story Types in the U.S.

|  | Past | Future |
| --- | --- | --- |
| Survival Values | 395/1001 (39%) | 223/1001 (22%) |
| Self-expression Values | 94/1001 (9%) | 267/1001 (27%) |
| Us versus Them | 311/1001 (31%) | 75/1001 (7%) |

**Online Appendix C**

Table C1. Five central national-story components and their incidence in each country

| Country | Past | Future |
| --- | --- | --- |
| Denmark | 1. World War II (13.96%)  2. Introduction of the Basic Law (13.86%)  3. Women's rights (7.43%)  4. Getting\losing Southern Jutland (5.45%)  5. Joining the EU (4.65%) | 1. Immigration policy  (10%)  2. A better welfare system (7.62%)  3. Employment opportunities (7.33%)  4. Environmental policy (6.63%)  5. Strong economy (5.05%) |
| Netherlands | 1. World War II (16.85%)  2. Independent state (4.35%)  3. 80 Years' War (3.52%)  4. Suffrage (3.31%)  5. Golden age (3.04%) | 1. More tolerant society  (18.72%)  2. Economic growth (6.35%)  3. Stay in or strengthen EU (5.80%)  4. Better politics/government (5.04%)  5. Solve the economic crisis (4.83%) |
| United Kingdom | 1. World War II (29.24%)  2. World Wars (4.49%)  3. Signing the Magna Carta (4.19%)  4. Women's rights (4.19%)  5. Birth of the NHS (3.69%) | 1. Exiting the EU  (8.58%)  2. Immigration policy (8.38%)  3. Strong economy (5.49%)  4. Equality (5.39%)  5. Optimistic future (3.59%) |
| United States | 1. September 11 attacks  (15.98%)  2. Freedom, rights, equality (8.79%)  3. Civil War (7.29%)  4. Independence (6.99%)  5. Revolutionary War (6.09%) | 1. Better economy  (9.79%)  2. Freedom, rights, equality (7.49%)  3. Peace (7.19%)  4. Election results (5.59%)  5. Social harmony (5.29%) |

*Note*. The numbers in parentheses represent the frequency of each story divided by the total number of past/future events in the respective country.

**Online Appendix D**

**Operationalization of Control Variables**

*Populist attitudes*. To what extent do you agree or disagree with the following statements? (Items presented in random order; for all items, 1=Strongly disagree and 5=Strongly agree; Items 5-8 were reverse-coded; scale reliability is between .64 and .76, depending on the country.) (1) Political parties are interested in my vote, not in my opinion; (2) Politicians do not understand what is going on in society; (3) There is a big gap between citizens and politics; (4) It doesn't matter whom you vote for, the situation remains the same; (5) Election campaigns give me enough information to make a choice; (6) The political candidates debated in an open and sincere campaign; (7) Parties can make a difference; (8) My friends and I have an influence on government policy. For each country separately we conducted principal component analysis and subsequent tests. Our analysis suggests that a two-dimensional summary of the eight statements is very effective. As is clear from the left-hand plots, for the most part, statements 1-4 are loaded together in one factor, and the other statements are loaded in another. We consequently control for these two factors.

|  |  |
| --- | --- |
|  |  |
|  |  |

*Note*. Principal Component Analysis –Populist statements. Right panel: scree plots of the eight PCs and a parallel analysis for the eight statements .Left panel: Factor loadings for the eight statements in the first two PCs.

*Political knowledge*. The number of correct answers a respondent could provide to two factual questions: (1) Can you name the Speaker of the Parliament? (2) Can you name the current Secretary of State?

*Political interest*. How interested in politics would you say you are? 0=Not at all interested, 10=Very interested.

*Left-right ideological self-placement*. We hear a lot of talk these days about liberals and conservatives. Below is a scale of the political views that people might hold, arranged from extremely liberal to extremely conservative. Where would you place yourself on this scale? 0=Extremely liberal, 10=Extremely conservative.

*Gender*. Are you male of female? 1=Male, 2=Female.

*Age group*. Which of the following age bands are you in? 1=18–24, 2=25–34, 3=35–44, 4=

45–54, 5=55+.

*Education*. 1=8th Grade or Less, 2=Some High School, 3=Graduated High School, 4=Trade or Technical School, 5=Some College, 6=Graduated College, 7=Post-graduate.

*Social class*. What social class would you consider yourself? 1=Upper class, 2=Upper middle class, 3=Lower middle class, 4=Skilled working class, 5=Working class.

*Income*. Thinking back over the last year, what was your annual household income before taxes? 1=Less than $5,000, 2. $5,000 to $7,499, 3. $7,500 to $9,999, 4. $10,000 to $12,499, 5=$12,500 to $14,999, 6=$15,000 to $19,999, 7=$20,000 to $24,999, 8=$25,000 to $29,999, 9=$30,000 to $34,999, 10=$35,000 to $39,999, 11=$40,000 to $49,999, 12=$50,000 to $59,999, 13=$60,000 to $74,999, 14=$75,000 to $84,999.

*Living in a rural region*. Where do you live? 1=In a rural area, 0=Any other answer (e.g., In a city with 50,001-500,000 inhabitants, In a city/town with over a 1,000,001 inhabitants).

*Table D1*. Descriptive Statistics.

|  | Denmark | Netherlands | UK | US |
| --- | --- | --- | --- | --- |
| Populist Attitudes (factor#1) | 3.9 (.89) | N.A | 3.9 (.8) | 3.7 (.8) |
| Populist Attitudes (factor#2) | 2.94 (.82) | N.A | 3.06 (.85) | 3.25 (.7) |
| Political Knowledge | 1.29 (.75) | .78 (.24) | 1.18 (.8) | 1.31 (.84) |
| Political Interest | 6.23 (2.65) | .54 (.28) | 6.85 (2.66) | 7.06 (2.91) |
| Ideological Self-placement | 5.06 (2.78) | 5.42 (2.13) | 5.56 (2.15) | 5.57 (2.68) |
| Gender (female) | 51.6% | 42.1% | 51.5% | 51.1% |
| Age | 49.22 (15.7) | 51.89 (15.88) | 47.45 (17.12) | 3.34 (1.48) |
| College degree (% of sample) | 21.8% | 27.9% | 35.3% | 32.8% |
| Rural (% of sample) | 15% | 12.1% | 18.6% | 13.4% |
| Upper class (% of sample) | 15.6% | 5.7% | 14.2% | 3.5% |
| Upper middle class (% of sample) | 51.5% | 26.2% | 33.8% | 24.85% |
| Lower middle class (% of sample) | 24.75% | 45.58% | 22.6% | 34.38% |
| Skilled working class (% of sample) | 8.12% | 22.6% | 29.4% | 37.2% |

*Note.* Entries are mean scores, standard deviations in parentheses. For gender we present the percentage of women in the sample.

**Online Appendix E. Support for the populist radical right as a function of story type, Multinomial Models**

Table E1. Support for the populist radical right as a function of story type (Multinomial Model, UK)

|  | (1) | (2) |
| --- | --- | --- |
|  | Populist radical right | Mainstream Right |
| *Boundary* | 0.93^***^ | 0.23 |
|  | (0.21) | (0.19) |
| *Survival* | -0.51 | 0.39 |
|  | (0.27) | (0.23) |
| *Self-expression* | -1.08^**^ | -0.05 |
|  | (0.39) | (0.29) |
| Populist attitudes (factor#1) | -0.17^***^ | -0.84^***^ |
|  | (0.19) | (0.16) |
| Populist attitudes (factor #2) | 0.96^***^  (0.18) | 0.31  (0.17) |
| Female | 0.15 | 0.29 |
|  | (0.30) | (0.26) |
| Political interest | 0.03 | -0.11 |
|  | (0.07) | (0.06) |
| Political Knowledge | -0.08  (0.19) | 0.11  (0.17) |
| L-R ideology | 0.58^***^ | 0.75^***^ |
|  | (0.08) | (0.08) |
| Rural | 0.65 | 0.78^*^ |
|  | (0.39) | (0.35) |
| Education | -0.19 | -0.23 |
|  | (0.21) | (0.18) |
| Class | -0.01 | -0.45^**^ |
|  | (0.15) | (0.14) |
| Age | 0.00 | 0.02^*^ |
|  | (0.01) | (0.01) |
| Constant | -6.61^***^ | -1.66 |
|  | (1.56) | (1.35) |
| N | 563 | 563 |

Note. Standard errors in parentheses; *** p<0.001, ** p<0.01, * p<0.05, + p<0.1. The Table reports results of multinomial regressions. Dependent variable is vote (party families) with vote for the mainstream left as a reference category.

Table E2. Support for the populist radical right as a function of story type (Multinomial Model, NL)

|  | (1) | (2) |
| --- | --- | --- |
|  | Populist radical right | Mainstream Right |
| *Boundary* | 1.27^***^ | -0.16 |
|  | (0.29) | (0.23) |
| *Survival* | -0.65^*^ | 0.01 |
|  | (0.25) | (0.19) |
| *Self-expression* | -1.01^**^ | 0.13 |
|  | (0.37) | (0.22) |
| Female | -0.39 | 0.31 |
|  | (0.36) | (0.25) |
| Political Knowledge | -0.31 | 1.12 |
|  | (0.80) | (0.66) |
| Political Interest | 0.80 | 0.80 |
|  | (0.69) | (0.53) |
| L-R ideology | 1.06^***^ | 1.22^***^ |
|  | (0.10) | (0.08) |
| Rural | 0.26^*^  (0.12) | -0.30^*^  (0.10) |
| Education | -0.36^***^ | 0.18^*^ |
|  | (0.11) | (0.08) |
| Class | 0.02 | 0.14 |
|  | (0.15) | (0.11) |
| Age | 0.00 | 0.03^**^ |
|  | (0.01) | (0.01) |
| Constant | -5.55^***^ | -11.45^***^ |
|  | (1.47) | (1.18) |
| N | 806 | 806 |

Note. Standard errors in parentheses; *** p<0.001, ** p<0.01, * p<0.05, + p<0.1. The Table reports results of multinomial regressions. Dependent variable is vote (party families) with vote for the mainstream left as a reference category.

Table E3. Support for the populist radical right as a function of story type (Multinomial Model, DK)

|  | (1) | (2) |
| --- | --- | --- |
|  | Populist radical right | Mainstream Right |
| *Us/Them* | 0.68^**^ | 0.06 |
|  | (0.21) | (0.21) |
| *Survival* | -0.54^*^ | 0.02 |
|  | (0.22) | (0.22) |
| *Self-expression* | -0.55 | -0.39 |
|  | (0.28) | (0.27) |
| Populist attitudes (factor#1) | 0.41^*^  (0.17) | 0.09  (0.16) |
| Populist attitudes (factor#2) | 0.35  (0.19) | 0.11  (0.19) |
| Female | -0.18 | -0.10 |
|  | (0.28) | (0.27) |
| Political interest | -0.08 | -0.04 |
|  | (0.07) | (0.07) |
| Political Knowledge | -0.22  (0.20) | -0.13  (0.21) |
| L-R ideology | 0.53^***^ | 0.60^***^ |
|  | (0.06) | (0.06) |
| Rural | -0.02 | 0.28 |
|  | (0.36) | (0.36) |
| Education | -0.40^***^ | -0.02 |
|  | (0.11) | (0.10) |
| Class | 0.02 | -0.86^***^ |
|  | (0.36) | (0.21) |
| Age | 0.01 | 0.01 |
|  | (0.01) | (0.01) |
| Constant | -4.54^***^ | -2.09 |
|  | (1.35) | (1.35) |
| N | 587 | 587 |

Note. Standard errors in parentheses; *** p<0.001, ** p<0.01, * p<0.05, + p<0.1

The Table reports results of multinomial regressions. Dependent variable is vote (party families) with vote for the mainstream left as a reference category.

**Online Appendix F**

Table F1. Marginal effect of *Boundary story* on the vote for populist radical right parties (logit Models)

|  | (1) | (2) | (3) | (4) | (5) | (6) | (7) | (8) |
| --- | --- | --- | --- | --- | --- | --- | --- | --- |
|  | UK | UK | US | US | DK | DK | NL | NL |
|  | Social class | Education | Social class | Rural | Education | Social class | Rural | Education |
| *Boundary* | 1.23^**^ | 1.11^***^ | 0.35 | 0.91^*^ | 1.22^***^ | 1.78^**^ | 1.58^*^ | 0.99 |
|  | (0.37) | (0.33) | (0.43) | (0.43) | (0.29) | (0.61) | (0.66) | (0.56) |
| Social class | -0.08 | 0.04 | -0.09 | 0.07 | 0.29 | -0.11 | -0.03 | -0.03 |
|  | (0.16) | (0.04) | (0.10) | (0.08) | (0.15) | (0.18) | (0.12) | (0.12) |
| *Boundary**class | -0.19^*^ |  | -0.03^**^ |  |  | -0.39^**^ |  |  |
|  | (0.09) |  | (0.01) |  |  | (0.14) |  |  |
| Education | -0.05 | -0.10 | 0.02 | -0.00 | -0.35^***^ | -0.45^***^ | -0.41^***^ | -0.42^***^ |
|  | (0.17) | (0.20) | (0.07) | (0.06) | (0.09) | (0.09) | (0.09) | (0.11) |
| *Boundary**education |  | -0.22^**^ |  |  | -0.29^*^ |  |  | -0.13^**^ |
|  |  | (0.10) |  |  | (0.12) |  |  | (0.04) |
| Rural | -0.00 | 0.09 | 0.12^*^ | 0.22^**^ | -0.18 | -0.17 | -0.57 | -0.51 |
|  | (0.30) | (0.27) | (0.07) | (0.08) | (0.30) | (0.30) | (0.75) | (0.76) |
| *Boundary**rural |  |  |  | -0.11^*^ |  |  | -0.60^*^ |  |
|  |  |  |  | (0.07) |  |  | (0.29) |  |
| *Survival* | -0.57^*^ | -0.60^**^ | -0.18 | -0.24 | -0.51^**^ | -0.52^**^ | -0.40^*^ | -0.39^*^ |
|  | (0.22) | (0.21) | (0.16) | (0.15) | (0.19) | (0.18) | (0.19) | (0.19) |
| *Self-expression* | -0.99^**^ | -0.55 | -0.66^***^ | -0.75^***^ | -0.32 | -0.35 | -0.92^**^ | -0.92^**^ |
|  | (0.36) | (0.29) | (0.18) | (0.17) | (0.25) | (0.25) | (0.34) | (0.34) |
| Populist attitudes | 0.86^***^ | 0.87^***^ | -0.00 | 0.00 | 0.55^**^ | 0.53^**^ |  |  |
|  | (0.20) | (0.18) | (0.14) | (0.13) | (0.18) | (0.17) |  |  |
| Female | -0.05 | -0.09 | 0.12 | 0.10 | 0.04 | -0.01 | -0.38 | -0.37 |
|  | (0.24) | (0.22) | (0.19) | (0.17) | (0.23) | (0.23) | (0.29) | (0.29) |
| Political interest | 0.10^*^ | 0.09^*^ | 0.08^*^ | 0.10^**^ | -0.04 | -0.04 | 0.96 | 0.95 |
|  | (0.05) | (0.05) | (0.04) | (0.03) | (0.05) | (0.05) | (0.55) | (0.55) |
| L-R ideology | 0.20^**^ | 0.20^***^ | 0.34^***^ | 0.34^***^ | 0.31^***^ | 0.30^***^ | 0.40^***^ | 0.40^***^ |
|  | (0.06) | (0.05) | (0.04) | (0.04) | (0.05) | (0.05) | (0.07) | (0.07) |
| Age | -0.00 | -0.01 | 0.17^**^ | 0.15^**^ | 0.01 | 0.01 | -0.01 | -0.01 |
|  | (0.01) | (0.01) | (0.06) | (0.06) | (0.01) | (0.01) | (0.01) | (0.01) |
| Constant | -6.19^***^ | -6.23^***^ | -3.93^***^ | -4.59^***^ | -5.23^***^ | -3.57^**^ | -2.49^*^ | -2.28^*^ |
|  | (1.17) | (1.07) | (0.86) | (0.79) | (1.16) | (1.09) | (1.11) | (1.09) |
| N | 752.00 | 878.00 | 704.00 | 831.00 | 696 | 696 | 1247 | 1247 |

*Note*. Standard errors in parentheses; *** p<0.001, ** p<0.01, * p<0.05, + p<0.1. The Table reports results of regression models predicting the vote for populist parties/leaders, interacting the Us/Them story type with socio-demographic variables.

Figure F1. Estimated effect of the Boundary story on the vote for populist radical right parties

*Note*. Marginal effect of the *Boundary* story on the vote for populist radical right parties (vertical axis) across levels of different socio-demographic variables. Marked are 95% confidence intervals. All moderating variables (on the x axis) span from marginalized groups to more privileged voters: The social class variable spans from working to upper class; education spans from 8th grade or less to graduate and post graduate; and Urban/Rural spans from living ln a rural area to living in a city/town with over 1,000,001 inhabitants. Results are based on estimation reported in Table F1.
