# Supplementary material for: Voices from the margins: How national stories are linked with support for populist radical right parties
Source: PLoS One. 2024 Aug 12;19(8):e0305554. doi: 10.1371/journal.pone.0305554 (PMC11318891; doi:10.1371/journal.pone.0305554)
Supplement: Attachment — Submitted filename: memo_plos1.docx [file pone.0305554.s003.docx]

Dear PLOS ONE Reviewers and Editorial Team,

Thank you for the invitation to revise and resubmit our paper, “Voices from the margins: How national narratives are linked with support for populist radical right parties” (Ms. No. PONE-D-23-23023). We thank the editor and the reviewers for their thoughtful and constructive comments, which have prompted us to significantly revise the paper. In the current version of the manuscript, we have expanded our discussion on national narratives and clarified how they differ from policy positions. We have also added a discussion on nativism and how it relates to national narratives. We have cross-referenced our classification of party-families with that of the PopuList dataset. We have revised the presentation of results, conducted several robustness checks, and bolstered confidence in our findings by excluding the populist attitudes as a control variable.

Below we present each reviewer’s comments in italics, followed by a discussion of how we address them in the revised manuscript. We are grateful for your thoughtful engagement with our research and hope you will now find the paper appropriate for publication in PLOS ONE.

*Response to Reviewer 1*

Reviewer 1 noted that "the paper presents an interesting and potentially novel idea." The reviewer added that s/he "liked the idea of testing the effect of ‘narratives’ (called here ‘national stories’) on the populist vote," and "thinks that there is much potential in this paper." The reviewer also raised some important questions and offered helpful suggestions for improving the work. We thank the reviewer for his/her support and constructive feedback, which allowed us to significantly improve the manuscript.

1.1 *"Regarding their research design, the authors propose three research questions but can only answer (and hypothesize on) two."*

Reviewer 1 is quite right, and we have revised the manuscript accordingly. In response to the comment, we have streamlined our research questions to two instead of three. The research questions are now as follows:

(a) What characterizes national stories held by populist radical right voters?

(b) What kind of voters are more affected by these stories?

We now better explain in the text how we address these two questions. To address the first question, we begin by providing descriptive association figures (Figure 1 a-d) illustrating the spatial proximity between stories and vote choice. The four graphs in Fig 1 represent the four countries in our sample and all highlight the separate positioning of mainstream and populist parties (or leaders). Next, through a series of regression models, we demonstrate that individuals embracing an “us versus them” *Boundary* narrative are more inclined to vote for populist radical right parties (PRRPs), whereas those embracing the other two types of narrative (*Survival* and *Self-expression*) are more likely to support mainstream parties.

Together, the aforementioned empirical analyses help provide an answer to our first research question. To address the second research question, we ran interaction models, which revealed that individuals we classify as “powerless” (i.e., low-educated, residing in rural areas, and with low social status) are more prone to translating the *Boundary* type of story into votes for populist parties compared to their more affluent counterparts.

1.2 *In their research design and results, the authors argue that there are significant differences in the national stories embraced by voters of PRRPs compared to those of mainstream party supporters (introduction, pages 3-4). According to their findings, individuals who endorse 'boundary national stories' are more inclined to vote for PRRPs. However, it is important to consider the possibility of overfitting if 'boundary national stories' are exclusively associated with an 'us versus them' narrative. If populist parties are the primary proponents of the 'us versus them' discourse, those who align with this idea are more likely to support populist parties.*

We appreciate the reviewer's comment, which prompted us to clarify the study’s theoretical foundations and the rationale for our primary dependent variable, the national story, in order to address concerns about potential overfitting or conflating national stories with policy agendas. We now elaborate in the paper that, while it is correct that populist radical right parties promote an “us versus them” rhetoric, they focus mainly on current politics. National stories, on the other hand, are least of all about current political affairs (see Table 1). They weave together the nation’s past historical events with future aspirations regarding it, and effectively constitute a blueprint for how people perceive their nation. Thus, theoretically, the construct of national story reflects a very broad perception of the nation – which, we contend, and empirically show, is a predictor of vote choice (p. 11).

Furthermore, from an empirical standpoint, if *Boundary* national stories were linked exclusively to the agenda and rhetoric of populist radical right parties, we would expect a perfect alignment between holding such a story and voting for this kind of party. However, our analysis (Fig 3) demonstrates that this is not the case. *Boundary* national stories are also prevalent among voters for mainstream parties on both the right and the left side of the political spectrum.

1.3 *Still related to their research design, the authors use two questions to gauge "national stories." One is related to past events ("Which past event do you consider to be the most important?"), and the other is related to future events ("What future event do you wish to see?"). The authors claim that the responses to these open-ended questions can be used as proxies for, among others, national stories that are deeply rooted in "in-group favoritism" and "out-group hatred." However, this does not seem to be the case from the examples and results provided in the draft. There is no further explanation or even description of how answers such as "World War 2" can relate to this "us versus them" narrative/feeling.*

We thank the reviewer for prompting us to elaborate on our coding decisions and to clarify the ways we coded past and future story components. Following this comment, we now expand on our coding scheme, and coding classifications. On pages 17-18 we now write:

*Boundary* national stories establish a division between "the people," or the national community – “us” – and everyone outside it – “them.” These stories inherently position other nations as outgroups, emphasizing a separation between them and the ingroup (the nation). The “us-them” boundary is thus a site for conflict between the national community and the outgroups. According to our coding scheme, responses relating to conflicts and clashes between the nation and an outgroup were coded as *Boundary*.

*Survival* stories, on the other hand, center around the nation’s economic and physical security and incorporate events and issues such as "the Industrial Revolution" and "Napoleonic wars" for the past, and "job opportunities" and "dealing with terrorism" for the future. Some events or issues lend themselves to a classification as both *Survival* and *Boundary*, and were accordingly relegated to both rubrics. For instance, narratives about wars or conflicts, such as "World War II," are salient to the nation’s physical security while at the same time marking its separation from other nations. Such elements were entered under both the *Survival* and the *Boundary* rubrics.

*Self-expression* stories revolve around human rights, democracy and the quality of life with stories such as “women’s rights,” “peace,” "signing the Magna Carta," "suffrage," and "freedom" are coded as such. A detailed explanation of the coding implemented, along with intercoder reliability scores and descriptive statistics for the three story types, are provided in Appendix B.

1.4 *Theory-wise, the draft is based on concepts such as "populism" and "radical right," since it focuses on "populist radical right parties." However, it does not define either of these concepts.*

Thank you for bringing this to our attention. We now include a definition of radical right populist parties in the introduction:

This group of parties adhere to an ideology characterized by authoritarianism, nativism, and populism (Mudde 2007). They take radical positions on cultural issues with nativism, a combination of nationalism and xenophobia, being central to their ideology. They are regarded as populist, as they profess to represent the will of the people, as opposed to that of a putative “other,” commonly depicted as a corrupt and self-serving elite (Margalit 2019).

Moreover, in Section 3 ("National stories and political behavior") we added a paragraph on nativism, a key feature in the ideology of populist radical right parties.

A key feature of the PRRP ideology is nativism, a belief that "states should be inhabited exclusively by members of the native group (“the nation”) and that nonnative (or “alien”) elements, whether persons or ideas, are fundamentally threatening to the homogeneous nation state" (Mudde 2010: 1173 ). Indeed, the distinction between “us” and “them” is at the ideological core of populism and is touted by populist leaders, who shift all blame from “the innocent people” to either out-groups or “corrupt elites.” Notably, however, while advocating an” us vs. them” narrative, populist parties focus mainly on current politics. Stories, on the other hand, are least of all about current politics: They weave together the nation’s past events with future aspirations regarding it, and as such effectively constitute a blueprint for how people perceive their nation. Rooted in a very broad perception of the nation, a national story reflects the national identity of the group that embraces it. Accordingly, a national story does not deal with current policy and occurrences, which preoccupy political parties.

1.5 *Data/method-wise, there are many inconsistencies along the draft. A few examples: variables present in some models but absent in others without explanation and variables presented during the text but not used in any of the models.*

Thank you for bringing this to our attention. We have ironed out inconsistencies throughout the paper. First, we reran our regressions and regenerated our coefficient plots. Now, all the control variables reported in the text (and only these variables) appear in our regressions and coefficient plots. Specifically, we write in the text:

The attitudinal and demographic variables we controlled for were derived from prior research on populist voting. The attitudinal covariates are populist attitudes, political knowledge, political interest, and ideological self-placement. The demographic variables are gender, age, education, social class, and a dummy variable for living in a rural region. The operationalization of all control variables, as well as their descriptive statistics, per country, can be found in Appendix D.

Consequently, we re-generated Fig 2 to include all control variables described above. Note that questions tapping populist attitudes are missing from the survey in the Netherlands, and thus the populist attitudes scale is not included in the figure on the Netherlands (the populist attitudes scale is also removed from the analysis as part of our robustness tests, see Fig F1 in the Appendix).

Second, in the previous version of the text, to test H2, we interacted social demographic variables with the *Boundary* story and reported the results in Fig 4. Fig 4 includes only the socio-demographic variables for which we got substantive and statistically significant results for the effect of the interaction term (socio-demographic variable * *Boundary* story). Due to space limitation, we chose to display in Fig 4 only substantive and significant results on which we had reported. Following the reviewer's comment about inconsistencies, as well as a similar comment made by R2, we added to the Appendix the full results of this empirical exercise. The graph below includes interaction terms between all the socio-demographic attributes of powerlessness and the *Boundary* story (see Appendix in the revised version). No statistically significant results were obtained for the urban/rural divide in the UK; for social class in the Netherlands; or for education in the US and Denmark.

1.6 *Also, the authors utilize Laver, Gallagher, and Mair (2011) and the Parlgov to classify the parties as populist (PRRP) or not, but do not provide any further explanation. This becomes problematic when Parlgov does not explain how they classify a party as "populist" or "populist radical right." In this sense, a lack of transparency and reproducibility is concerning.*

Following the reviewer’s comment, we now explain in the text how we use these data sources to classify parties to party-families. Also, we cross-reference our categorization with the PopuList classification of radical right populist parties. There was a perfect match between our sources for classification of PRRPs and the PopuList’s. See [here](https://popu-list.org/wp-content/uploads/2023/09/Denmark.pdf), for the case of Denmark; [here](https://popu-list.org/wp-content/uploads/2023/09/Netherlands.pdf) for the classification of the PVV in the Netherlands as PRRPs, and [here](https://popu-list.org/wp-content/uploads/2023/09/United-Kingdom.pdf) for the classification of the UKIP in the UK; The US is not included in the PopuList dataset. We added a footnote regarding this alignment of our classification to that of the PopuList:

Footnote # 2: Our classification of populist radical-right parties is also consistent with the [PopuList](https://popu-list.org/applications/) classification (Rooduijn et al., 2023), which is based on country-specific experts’ and comparativists' assessment of these parties' core ideological attributes.

*Response to Reviewer 2*

Reviewer 2 stated that this was a “an exciting article that measures the impact of national narratives on voting for populist radical right parties or candidates.” At the same time, s/he offered several helpful suggestions for improving the manuscript. We are grateful for these insights and have worked diligently to implement the reviewer’s advice.

2.1 *The authors refer to “national stories” that draw the borders of us versus them populist identities. However, if the authors talk about “national stories” or “national past,” then they focus on right-wing populist parties rather than left-wing ones. Therefore, there should be more discussion about nativism than populism. As Mudde (2007) argues, the populist radical right has three characteristics: nativism, populism, and authoritarianism. Also, it is nativism rather than populism that is the most prominent characteristic of these parties and candidates.*

We thank the reviewer for pointing this out. We now clarify in the text that we focus on right-wing rather than left-wing populism. We thus also changed the title of the paper to include the words "radical right" in addition to "populist." We also added a discussion on the three core features of right-wing populism: nativism, populism, and authoritarianism; and as part of the discussion leading up to our first hypothesis, we state the expected relationship between nativism as an ideology and an “us versus them” *Boundary* story. We consequently cite Mudde 2007, 2011. On pages 10-11 we now write:

Our first theoretical expectation emanates from previous works on the importance of narratives, and specifically, on the idea that national stories reflect individuals’ political identities. If so, we expect to find differences between stories embraced by voters for mainstream and populist parties. Moreover, the demand side literature describes voters for PRRPs as having reverted to nativist values of exclusion and discrimination, in opposition to elites’ global and universalistic values (Mudde and Kaltwasser 2012), and as tending to glorify the past (Elçi 2022). Previous works claim that right-wing populist parties mobilize potential voters by fueling feelings of resentment and anger tapping on their outsiderness. These studies point at populist actors’ tendency to see the world in terms of us-versus-them dichotomies, be it “the people” versus the “corrupt elite” or negative sentiments toward out-groups. A key feature of the PRRP ideology is nativism, a belief that "states should be inhabited exclusively by members of the native group (“the nation”) and that nonnative (or “alien”) elements, whether persons or ideas, are fundamentally threatening to the homogeneous nation state" (Mudde 2010: 1173). Indeed, the distinction between “us” and “them” is at the ideological core of populism and is touted by populist leaders, who shift all blame from “the innocent people” to either out-groups or “corrupt elites.”

We also added these three features to the description of populist radical right parties in the introduction:

Populist radical right parties (PRRPs) have become a significant force in politics over the last few decades, joining and even leading governments in a growing number of countries (Akkerman et al. 2016; Berman 2021; Noury and Ronald 2020). This group of parties adhere to an ideology characterized by authoritarianism, nativism, and populism (Mudde 2007). They take radical positions on cultural issues with nativism, a combination of nationalism and xenophobia, being central to their ideology. They are regarded as populist, as they profess to represent the will of the people, as opposed to that of a putative “other,” commonly depicted as a corrupt and self-serving elite (Margalit 2019). Such parties have significantly impacted policy-making, and in certain cases, they have eroded democratic norms and institutions.

2.2 *The authors did not explain the case selection. There should be at least a few sentences about why the authors examined Denmark, the Netherlands, the UK, and the US.*

We have corrected this omission by adding a paragraph to the “Data and measurement" sub-section, explaining our case selection. Specifically, we wrote:

The study tests the relationship between populist voting and national stories in four countries with different histories – notably, with respect to immigration and PRRP support: Denmark, the Netherlands, the United Kingdom and the United States. These countries were chosen also because they are all developed democracies, but differ in their political systems as presidential vs. parliamentary democracies, as well as their electoral systems and district magnitude (e.g., extreme proportionality in the Netherlands vs. single-member plurality in the UK and the US). The above variations across the countries sampled make it possible to extrapolate the results to other developed democracies and draw general inferences regarding the relationship between national stories and vote choice.

*In the introduction (p. 4), the authors referred to the populist constituency as “the bottom of a society’s power structure.” On p. 12, they said that “marginalized people might cope with their subordinate position and at the same time strive to gain greater acceptance to mainstream society by distancing themselves, physically or psychologically, from other marginalized groups, and labeling the latter as outsiders and a threat to the community.” In short, the authors refer to the relative deprivation argument in the broadest sense rather than economic grievances. They also used varying sociodemographic variables in interaction models. Thus, it would be better to focus on relative deprivation literature more than losers of globalization. For additional literature, you can check societal pessimism (Steenvoorden & Harteveld, 2018), nostalgic deprivation (Gest et al., 2018), and collective nostalgia (Elçi, 2022).*

Thank you for pointing to additional literature salient to our study. Following the reviewer’s suggestions, we now discuss the relative deprivation argument and engage with the relevant literature. As part of our discussion on research that combines economic and cultural explanations to populism, we write the following:

A growing number of scholars have used insights from both the cultural and the economic approaches to construct more complex, but potentially more causally convincing, accounts of populism (Mutz 2018; Stockemer et al. 2018). Some of these explanations link changing economic conditions to right-wing populism via status anxiety: A decline in the social standing and the growing marginalization of manual workers have created a fertile ground for a politics of collective status-threat that mobilized voters leveraging their deep resentments toward professional elites and minorities (Bonikowski 2017; Gidron and Hall 2020). In this context, Guiso et al. (2017) document a link between economic insecurity and distrust in political parties, on the one hand, and voting for populist parties, on the other. Algan et al. (2017) study the political consequences of the Great Recession in Europe, and find that post-2008 elections show the sharpest decline in trust in institutions and establishment politics in regions where unemployment had increased. Steenvoorden and Harteveld (2018) demonstrate a link between support for PRRPs and societal pessimism, which cannot be explained based on objective conditions alone. Gest et al. (2018) show that support for the radical right in the UK and the US is driven by nostalgic deprivation, the subjective perception of status decline. In investigating the impact of individuals’ national stories on their vote choice, our paper builds on the above empirical literature that endeavors to marry the economic and cultural explanations for populism. The thrust of our investigation is to identify the various characteristics and factors that render one’s vote susceptible to such narratives, along with the features of a national story that increase the likelihood of populist leanings.

We also briefly discuss the work on collective nostalgia by Elçi (2022) in Footnote #1

An important study conducted in Turkey shows how nostalgia, the glorification of past history, is linked to different forms of populism in that country (Elçi 2022). Our paper is consistent with this work, with two caveats. First, we contend that a national story is not only about the past, but also incorporates a future outlook on the nation. We further claim that past and future story elements are equally important in gauging a voter's perception of their nation and in predicting their voting decisions in the present. Second, we submit that it is the Boundary type of national story that is linked to support for right-wing populism. Nostalgia may exacerbate in-group vs. out-group distinctions, but essentially it is glorification of the country's good old days.

2.3 *For selecting populist parties, please also check the PopuList dataset (Rooduijn et al., 2023). Nevertheless, it would be better to acknowledge that voting for populist parties is not equal to having populist attitudes. There can be populists who vote for mainstream parties for mechanical and psychological reasons, especially in the UK.*

Following the reviewer's valuable comment, we cross-reference our party classification with that of the PopuList dataset 3.0. Our classification of parties to PRRPs aligns perfectly with that of the PopuList, and we acknowledge this fact in Footnote #2 in the revised version of the paper. Also, we acknowledge in the text that voting for populist parties is not tantamount to having populist attitudes and call for further research on populist attitudes. In the Conclusion section we write:

Furthermore, research has shown that voting for populist parties does not always align with populist attitudes, and that some voters with populist views may choose mainstream parties for strategic or other reasons; accordingly, future research could explore the connection between national stories and populist attitudes. Our findings indicate that an “us versus them” *Boundary* story is prevalent not only among populist voters but also among those supporting mainstream parties. A follow-up study could provide a more nuanced understanding of the association between the “us versus them” *Boundary* story and populism, measured in terms of attitudes rather than voting choices.

As suggested by the reviewer in another comment, and by way of a robustness check, we removed from the analysis populist attitudes as a control variable (Figure F1 in the Appendix).

2.4 *The generation of national story variables is not clear. Is it a continuous scale or categorical? However, more importantly, what is the left-out group for stories? The authors used three of them in the same regression without having a multicollinearity issue. Please explain these issues in more detail.*

Following the reviewer's comment, we have expanded the explanation on how the three scales were generated (see page 18).

The score on the *boundary*, *survival* and *self-expression* scales was assigned based on the number of components a respondent provided for the respective story type. Thus, for example two *survival* components (one for the question about the past and one for the question about future prospect) earned the respondent the highest score on that scale (2), one component (past or future) earned the respondent a mid-score (1); while absence of such components was tantamount to the lowest score (0). The higher the score on a scale, the stronger the leaning towards the respective national story type. A similar score was produced for the other types of national stories (*boundary and* *self-expression)*.

Note that our categorization scheme does not encompass all story components cited by respondents, such that some were left uncategorized. Examples of story components that are assigned a zero score on all scales are as follows: In Denmark, "The Christianization of Denmark" or "Winning the EU football championship," both for the past; in the Netherlands, the "death of Prince Claus" for the past and "political stability" for the future; in the US, "the Hillary Clinton era," "Kennedy’s assassination," and "slavery" – all for the past. The proportion of this left-out group is 26% in Denmark, 22% in the Netherlands, 28% in the US, and 21% in the UK.

We also note in a footnote that the correlation between the three story scales is low to medium. We write the following:

Correlation between the three story scales is low to medium, and spans from .42 between the *boundary* and *survival* scales in Denmark to -.09 between the *survival* and *self-expression* scales in the UK.

2.5 *Figures 1 a-d should be presented before explaining the results to provide a better picture to the readers. Also, average marginal effects rather than log odds could be reported to understand better and compare the magnitudes of independent variables.*

This comment is important. We have restructured the Results section consistent with the reviewer’s suggestion, placing Fig 1 a-d earlier in the paper, before the explanation of the results. We also report substantive results concerning our main IV, the *Boundary* story. In the Results section, we now write:

The magnitude of the effect is substantial. In Denmark, the UK, the US and the Netherlands, moving from not citing either past or future *Boundary* story components to citing both such components increases the probability to vote for the right-wing populist party/leader by 20, 19, 20 and 15 percentage points, respectively

2.6 *The authors used different sociodemographic variables for interaction models. It is not easy to understand why they did not use the same ones in every country. Please explain how the socioeconomic variables were selected.*

Thank you for this comment. We now explain in the text that Fig 4 includes only the socio-demographic variables for which we got substantive and statistically significant results for the effect of the interaction term (socio-demographic variable * *Boundary* story). Due to space limitation, we chose to display in Fig 4 only substantive and significant results on which we had reported. Nevertheless, following the reviewer's comment, as well as a similar comment by R1, we added to the Appendix the full results of this empirical exercise. The graph below includes interaction terms between all the socio-demographic variables and the *Boundary* story (see Figure F2 in the revised Appendix). No statistically significant results were obtained for the urban/rural divide in the UK, for social class in the Netherlands, or for education in the US and Denmark.

2.7 *The authors did not explain the sampling and data collection processes. They should add a detailed explanation for each country either to the main text or the appendix.*

This information has now been added to Appendix A, and accordingly, in Footnote #2 we now ask the reader to refer to this addendum: "For details on survey companies, sampling techniques, and descriptive statistics of the samples, see Appendix A."

2.8 *Finally, as a robustness check, the authors could run the regressions without populist attitudes for the other three countries. In fact, they could also remove them from the analysis since they are not using a conventional populism battery.*

Following the reviewer’s suggestions, in Figure F1 in the revised Appendix we demonstrate that results hold even if we remove the populist attitudes scale from the analysis.
