# Supplementary material for: Voices from the margins: How national stories are linked with support for populist radical right parties
Source: PLoS One. 2024 Aug 12;19(8):e0305554. doi: 10.1371/journal.pone.0305554 (PMC11318891; doi:10.1371/journal.pone.0305554)
Supplement: Attachment — Submitted filename: PLOS ONE (R&R) (2024) Review.docx [file pone.0305554.s004.docx]

Comments to the authors (R&R)

I appreciate the effort and the changes made by the authors. Personally, I liked how the authors handled some of the comments regarding their research question, and I found that the changes improved heavily on the original manuscript. However, the third and fourth points remain problematic, and many (if not all) minor comments were left unaddressed. Since I believe this might be due to some problem that prevented them from accessing the file attached to the original review or maybe even an oversight, I recommend another round of revisions. I am attaching my original comments again.

Here are some other comments based on this round:

1)	Related to the research design, I am still not convinced about how the questions used for both past and future events can be used as proxies for national stories “deeply rooted in in-group favoritism and out-group hatred.” Even though the authors addressed this comment in their response, I am still unconvinced.

a.	“Boundary national stories establish a division between “the people,” or the national community – “us” and everyone outside it – “them.” These stories inherently position other nations as out-groups, emphasizing a separation between them and the in-group (the nation).” This type of story seems to be related to only nationalism. Both “national community” (treated as “us”) and “everyone outside it” (treated as “them”) seem to measure nationalism, as there is no reference to “elites” or the moral division between them and “the people,” or “us.” In this sense, if the authors are studying PRRPs (Populist Radical Right Parties), they will indeed find a relationship between “boundary national stories” and support for this party family. However, this connection will be driven mainly by nationalism and not populism.

b.	How would “Napoleonic wars” and “Industrial revolution” (as past events) gauge a “survival story”? Given how long it has been since both events, can you confidently affirm that they still provoke sentiments related to economic and physical securities? I do not think this is the case.

c.	How would “World War 2” mark the “separation from other nations”?

d.	Finally, do the terms “survival” and “self-expression” come from studies in political culture? They seem to resemble Inglehart’s definitions heavily. If that is the case, why not acknowledge that?

2)	There is still a problem with the concepts. My original comment was that the manuscript lacked a proper definition of concepts such as “populism” and “radical right” (consequently, “populist radical right”). The authors responded by adding a couple of phrases to the introduction and section 3. However, I do not think this is enough.

a.	First, there is still no proper definition of populism.

b.	Second, even though they added a couple of phrases in section 3, there needs to be a connection between “national stories” and “(radical right) populism.” What I mean by that is that the authors should properly connect these two things, developing how we should expect national stories to relate and connect to radical right populism.

c.	Third, I would suggest caution when writing that “These studies point at populist actors’ tendency to see the world in terms of us-versus-them dichotomies, be it “the people” versus the “corrupt elite” or negative sentiments toward out-groups” (p. 11). Since populism requires anti-elitism, this sentence might be misleading.

d.	Finally, I suggest splitting this paragraph (p. 11) into smaller parts.

3)	When talking about the three types of stories, the authors state that “while the first two kind (survival and self-expression) encapsulate the norms, goals, and values of the national group, the Boundary story defines the national community relationally and negatively, in terms of exclusion.”

a.	First, are the classifications of stories on “survival,” “self-expression,” and “us versus them boundary” mutually exclusive? Can boundary’s (us versus them) exclusion also be done through values and norms? Moreover, in this sense, would it still be “boundary” or something different?

b.	Second, how can “survival” and “self-expression” expressed in stories relate to both material and economic survival (or freedom and emancipation) and “norms, goals, and values” at the same time? An example: how can “Napoleonic wars” relate to survival, in terms of material and economic security, and “norms, goals, and values of the national group” simultaneously? This seems to be stretching or reading in too much.

4)	Have the authors tried using “stories” as moderators/mediators in their models? Although I am not entirely convinced yet of the importance of “stories” as predictors for support for Populist Radical Right parties, I think it makes sense to think of them as either moderators or mediators (depending on the authors’ theories and points of view).

5)	Finally, I would suggest removing the variable “populist attitudes” from the models. First and most importantly, these variables (Appendix D) do not measure populist attitudes. While items 1 to 3 could be used, to some extent, items 4 to 8 measure different things, among which political efficacy (which has been commonly mistaken or even incorrectly used as a proxy for populist attitudes). Second, there is no further information on how the scale was created. If the authors plan on keeping these variables as a measure of populist attitudes, I would recommend (1) calling it something different, (2) displaying the result of an EFA or a CFA (depending on whether this scale was used before), and (3) explaining how the variables were aggregated, or how they come together to form a scale.

Below is the rest of my initial comments.

Comments to the Author

The paper presents an interesting and potentially novel idea. I personally liked the idea of testing the effect of "narratives" (called here "national stories") on the populist vote, and I think there is much potential in this paper.

However, in the end, these are not adequately explored, and the authors overestimate their contribution. In general, some fundamental flaws make its publication not recommended. Namely, major problems with its research design, theory, and writing require substantial rework and reconsideration. Broadly speaking, these are:

1 – Regarding their research design, the authors propose three research questions but can only answer (and hypothesize on) two.

2 – In their research design and results, the authors argue that there are significant differences in the national stories embraced by voters of PRRPs compared to those of mainstream party supporters (introduction, pages 3-4). According to their findings, individuals who endorse 'boundary national stories' are more inclined to vote for PRRPs. However, it is important to consider the possibility of overfitting if 'boundary national stories' are exclusively associated with an 'us versus them' narrative. If populist parties are the primary proponents of the 'us versus them' discourse, those who align with this idea are more likely to support populist parties.

3 – Still related to their research design, the authors use two questions to gauge "national stories." One is related to past events ("Which past event do you consider to be the most important?"), and the other is related to future events ("What future event do you wish to see?"). The authors claim that the responses to these open-ended questions can be used as proxies for, among others, national stories that are deeply rooted in "in-group favoritism" and "out-group hatred." However, this does not seem to be the case from the examples and results provided in the draft. There is no further explanation or even description of how answers such as "World War 2" can relate to this "us versus them" narrative/feeling.

4 – Theory-wise, the draft is based on concepts such as "populism" and "radical right," since it focuses on "populist radical right parties." However, it does not define either of these concepts.

5 – Data/method-wise, there are many inconsistencies along the draft. A few examples: variables present in some models but absent in others without explanation and variables presented during the text but not used in any of the models.

6 – Also, the authors utilize Laver, Gallagher, and Mair (2011) and the Parlgov to classify the parties as populist (PRRP) or not, but do not provide any further explanation. This becomes problematic when Parlgov does not explain how they classify a party as "populist" or "populist radical right." In this sense, a lack of transparency and reproducibility is concerning.

Now, here are some specific comments and suggestions for improvement.

1 – Introduction:

- The introduction should be shorter. It takes some space from other sections. In this sense, the authors would significantly improve the flow of their paper if they cut or reworked their introduction.

- Research question 3: "What kind of voters are more affected by these stories?" From RQ3, it seems like the authors are interested in what kind of voter is more affected by boundary stories, however, in the sense as to measure the degree to which respondents are affected by something, I do not think observational data is enough.

- The authors present their typology for national stories (survival, self-expression, boundary). However, there is no further information on how these categories were created. I explain this in detail below, but it seems like it is an arbitrary decision.

- Throughout the introduction and pretty much the entire theory section, the authors state that the effects they are interested in are stronger for those positioned at the bottom of a [society's] "power structure" – making up for the "powerless." What defines someone as "powerless"? Not only that, but this term seems to give place to "marginalized" later on during the data and analysis sections. Although there is a relationship between powerlessness and marginalization, they represent different dimensions of social disadvantages. I suggest the authors pick and choose one of these terms or explain how they are used interchangeably, as it would significantly improve the overall flow of their narrative.

- The authors claim their results "challenge early studies that inferred electoral motives of voters in Western Democratic societies directly from their material life conditions." While I could think of a few papers that do this for populism, this is an affirmation that needs references.

- One of my main concerns is on the second paragraph, page 4. The authors claim that PRRPs supporters have distinct socio-structural attributes, including [being] "manual working class," citing Coffé (2018). Although this is, to some extent, true, as PRRPs supporters tend to be from the working class and blue collars, Coffé is talking about RRPs, not PRRPs. Again, although there is indeed an overlap between both, and in the 21st century RRPs are predominantly populists, a work that focuses exclusively on RRPs should not be used to describe the profile of PRRPs supporters – and if it used, this needs to be acknowledged and made explicitly clear. Second, and most importantly, Coffé's argument is that men are overrepresented in these jobs, which are the most threatened by modernization, globalization, and immigration; that is why they resonate more with RRPs. Leaving this explanation aside and focusing solely on the part of the "manual working class" to reinforce the argument that researchers have found distinctive social-structural attributes that predict the vote for PRRPs is misleading.

- Still related to that, when saying that PRRPs supporters have predominantly "low-to-intermediate levels of education," the authors use Becker et al. (2017) as a reference. I suggest using a different one. Becker et al. discuss Brexit, and although there are some overlaps between Brexit supporters and PRRP supporters, these should not be treated as "one and the same." Even though Becker et al.'s (2017) robustness tests compare Brexit supporters to the electorate of Marine Le Pen, I do not think it is enough. We know from extensive research and literature that PRRPs supporters tend to be in the "low-to-intermediate" levels of education, but I suggest using a different reference here, maybe Danieli et al. (2022).

- The introduction focuses on socio-structural attributes that distinguish PRRPs supporters from the rest. However, the authors miss the bigger picture by focusing on just socio-structural attributes. First, socio-structural attributes, such as those cited by the authors, are just one of many facets of supporting PRRPs. In this sense, most of the time, these are the consequences of other types of attitudes/feelings. An example, quoting the authors, would be how PRRPs garner support "mainly among the manual working class." Although this is correct in terms of socioeconomic structures, in reality, it is a consequence of a broader feeling of insecurity that manual/blue-collar workers face. As Coffé (2018) puts it, globalization and modernization make it more likely for them to lose their jobs, be forced into lower-paying jobs in the new global economy, or be substituted by immigrants. Therefore, economic grievances and insecurity can lead to (an anti-immigration) nativism, which would lead to Radical Right Parties' support. The draft would be greatly improved if the authors acknowledged this.

- Still related to that, one of the main critiques the authors make is that: "such ascriptive categories may not capture the social identities that individuals would deem relevant (…) [as] individuals strive for a positive self-concept and thus tend to construct their identities in more affirmatory terms" (p. 4). However, this is a conclusion driven by, what I think is, a misinterpretation of the profile of PRRPs supporters. As it is the case for many of the references the authors use, these "ascriptive categories" are rooted in much more complex feelings that range from insecurity and threat to a "silent counter-revolution." Therefore, it is my understanding that these categories are not made to be interpreted alone and that in their full contexts, they indeed capture social identities that individuals deem relevant (to use the authors' words).

- The authors state that "Criteria anchored exclusively in economic indices are also implausible since PRRPs, as a party family, do not present a coherent economic ideology or a unified stance regarding the first dimension of politics" (p. 4). I believe this is wrong. First, as part of the Right-wing party family, these parties do present, to some extent, a coherent set of economic ideologies/proposals. More evenly, as part of the far-right party family, even more distinct proposals distinguish them from the rest. In this sense, I am not sure if this lack of ideological consistency comes from the authors' interpretation of populism as a thin-centered ideology, but I believe the authors would greatly benefit from reading Mudde (2019). Second, most of the criteria anchored in economic indices (i.e., Berman, 2021) are also related to other explanations often associated with values and cultural priorities.

- There is no description of the methods the authors use to test their hypotheses or to answer their research questions. Then, on page 5, they say, "We empirically demonstrate how a motley slew of grievances find voice in populistic agendas, by being channeled into a unifying boundary narrative that affords stigmatized groups a positive distinctiveness." The authors do not demonstrate empirically how grievances find voices in "populistic agenda." This is unrelated to their research question and is not answered throughout the text. I suggest the authors rephrase or reformulate this phrase.

2 – Cultural and Economic Approaches to Study Populism

- My main concern regarding this section is the lack of any definition of populism. It is possible to understand from part of the literature the authors mobilize that they will approach populism from its ideational definition. However, it is not possible to be entirely sure of it, and the draft does not define populism in any way. More concerningly, there is no definition of (populist) Radical Right parties. In this sense, their paper does not define the central concept utilized and mobilized by the authors.

- Some minor comments: 1) the authors claim that "scholars have not been able to establish empirically consistent connections between individuals' economic circumstances such as income, wealth, or employment status and the propensity to support populism" (p. 6) and that their results will corroborate these outcomes. This is somewhat confusing. Are the results corroborating the lack of consensus or the relationship between individuals' economic circumstances and support for populism?

2) The subsequent phrase is tough to follow. "Second, it has been established that people tend to identify more with high- than low-status groups. Thus, support for populist causes cannot be fully, nor indeed directly, accounted for by socio-demographic marginality." It is difficult to follow the authors' reasoning here. Also, again, and risking being over-repetitive, I think the authors should consider that socio-demographic factors are rarely used as an actual explanation for populist success. Rather, you can use socio-demographic variables to trace a profile for the populist supporters or even the populist individuals (as the literature has used so far, e.g., Rovira Kaltwasser & Van Hauwaert, 2019), however, when it comes to electoral success of these populist parties, these socio-demographic variables are related to other types of values, feelings, and/or cultural priorities that drive the vote for the populists.

- On page 7, to strengthen the authors' argument in favor of approaches that combine cultural and economic explanations, I suggest they read Rico et al. (2017). It might not be a paper combining both approaches, but it provides interesting ways to think about it.

However, on second thought, since the authors do not actually engage with cultural explanations, I think they should restructure their section (I talk more about this below).

- Minor comment: Last paragraph, p. 7: "Guiso et al. (2017) document a link between economic insecurity and distrust in political parties, on the one hand, and voting for populist parties, on the other. Algan et al. (2017) study the political consequences of the Great Recession in Europe, and find that post-2008 elections show the sharpest decline in trust in institutions and establishment politics in regions where unemployment increased." The authors could link this to populism through the idea of how "declining trust" in political institutions and establishment can lead to support for populism. The way it stands now makes very little sense in the paragraph's narrative.

- Minor comment: At the end of this section, the authors claim that this paper aims to identify the various characteristics and factors that render one's vote susceptible to such narratives, along with the features of a national story that increase the likelihood of populist leanings. I would recommend rephrasing that. The way it currently is makes it very difficult to understand that the authors are referring to national narratives, not economic and cultural ones (the first half).

3 – National Stories and Political Behavior

- I recommend changing the title of this section and getting rid of "political behavior," as this section focuses very little on actual political behavior.

- "Populist ideas in general are anchored in national identity (…)" (p. 9). I do not know if I agree with that. If we think about the general idea of national identity, I would say this is not the case. Populist ideas, depending very much on the approach you adopt (I will assume the authors are talking about the ideational definition since it is the most commonly used in the study of populist ideas), are related to a specific populist potential among individuals, or in other words, the degree to which individuals are more susceptible to the populist discourse, or agree with what constitutes populism. In this sense, national identity has little to do with people-centrism and anti-elitism. It can serve as one way to bring the "us" together more easily and consequently equate it to "the people," but I would not go as far as to say that populist ideas are anchored in national identity.

- The terms "national identity," "national narrative," and "national stories" are used generically and loosely. This makes it difficult to properly understand what the authors mean when using one of these terms.

- Page 10, paragraph 2: "Previous works claim that populist parties mobilize potential voters by fueling feelings of resentment and anger and tapping on their outsiderness." I suggest the authors add a reference for that, especially when discussing "previous works."

- Hypothesis 1: What is a boundary story? The section leads to this hypothesis and gives some "hints" as to what we could expect from a boundary story, but never properly defines it.

4 – Why are some voters more sensitive than others to the boundary story?

- The authors explain that their answer for "what explains the difference in effect of the boundary story on vote choice" comes from economic grievances. However, they write early during the Introduction and Section 2 that their work would build on the "empirical literature that endeavors to marry the economic and cultural explanations for populism." Until this point, there are no references to how the cultural explanations would fit their research/would be used to answer their research questions. I suggest reevaluating this proposal. If cultural explanations are indeed important for their research design, I suggest rewriting the other sections to make them explicit. Otherwise, I would suggest to just drop it.

- 2nd paragraph, page 12: "We argue that the boundary story is likely to be more central to the self-definition of individuals stationed along an in-group's periphery, in terms of geography (far flung), education (medium and low) and status(low), and are in a sense marginalized."

What would be a "low status"? Is it in economic terms? Also, the authors argue that boundary stories are likely to be more central to individuals who, among many other things, are "in a sense marginalized." What does that mean? If this is one of the main "characteristics" they expect to find in their results, it should not be left as generic and vague. I suggest clarifying what constitutes a "marginalized" group and how to define/measure it.

- In general, the authors emphasize the success of populist parties and how narratives/stories could lead to support for populism. Therefore, I think the draft paper would greatly benefit from articles that also approach populism from a discursive perspective, especially those related to the success of populist parties. I suggest starting with papers that approach populism from a discursive approach, e.g., Hawkins (2009, 2010) and/or Poblete (2015). Also, a suggestion for the success of populist parties that might help the authors in their thinking is Castanho Silva (2018).

- Finally, I am not entirely sold by their argument on the relationship between national stories and "marginalized groups." For marginalized groups, boundary-national stories might have a more significant impact on their self-definition. However, it might as well be the case that these individuals present these boundary-national stories because they are marginalized. What I mean is that these two "variables" seem to be highly correlated, and given their importance to the paper, their multicollinearity cannot be ignored. I do not think the authors adequately address (or even acknowledge) that.

- Page 13, the authors state that "this hypothesis aligns with the economic grievances literature, arguing that socio-demographic attributes are important for populist support, but crucially adds the contention that the effect of these attributes on the vote is moderated by an us-versus-them boundary story." I do not think that this is the case of a moderation effect. I think that it is a mediation effect. If we start from the economic grievances, then socio-demographics are just as related to an "us-versus-them" thinking as it is to the populist vote.

5 – Empirical Strategy

- The data was collected online. However, how was the sample selected? Is the sample representative of the actual population of each one of these four countries?

- The authors mention that they also control for "nativist attitudes" (p. 13, 1st paragraph, Empirical Strategy). However, there are no other mentions of these attitudes in their paper or online appendix. Were nativist attitudes used at all? I suggest clarifying that.

- The authors mention that they control for "populist attitudes," but provide no further explanation as to how the scale was constructed, if the items load together (assuming they employ Factor Analysis techniques), or which literature was used as reference when creating their scale. This is very important because, given their aim and the model they propose, populist attitudes would be a confounder in the relationship between boundary national stories and the vote for PRRPs. I suggest clarifying this.

- The authors base their party classifications (PRRP, Mainstream Right-Left) on the work of Laver, Gallagher, and Mair (2011) and the Parlgov data (2016). Parlgov, however, does not provide a clear explanation as to how they classify a party as "populist" or even what they understand as "populist." In this sense, there is no way to evaluate the validity of the authors' classification or even know if the parties were properly classified. This is a major point to be addressed by the authors, especially praising transparency and reproducibility. Finally, if the authors plan on sticking to Laver, Gallagher, and Mair's and Parlgov's classifications, I suggest comparing them to Poppa, the PopuList, or even V-Dem's V-Party. These are more well-known classifications with clear criteria and descriptions and would work as a good type of "robustness test."

- National story is measured through two types of open-ended questions, one on past events and the other on future events. Given the original questions (p. 15) and the following phrase from the authors: "These two open-ended questions directly target the nation's past and future, and thus tap by proxy respondents' national stories," I have two comments:

(1) I am not entirely convinced that asking respondents about "which past event [they] consider to be the most important" and "what future event [they] wish to see" can work as a proxy to infer their national stories, let alone measure how respondents' "picture" their own nations (as the authors argument in p. 9).

(2) I do not think that by asking about past and future events (the way the questions were originally worded), the authors can infer the existence of an "us-versus-them" national story. Also, there are many reasons why someone might answer "World War 2" or "end of terror" for their past event question (using the same example as given by the authors), be it cultural, material, or more immediate reasons. In this sense, the authors do not convince that these can be used as proxies for "us-versus-them" national stories. A good exercise here would be to answer why and how these events can be related to "us-versus-them" national stories and, ideally, how they can lead to a populist (radical right) vote.

(3) Finally, one minor comment: asking just one past event and one future event might not be the best way to gauge respondents' national stories, as many external factors might contribute to their answers. Depending on things such as exposure, context, and so on, respondents might be inclined to give different responses that do not actually align with their "national story."

- Other minor comment: an intercoder reliability of 0.7 Is not necessarily problematic, but it should be noted that, according to Krippendorff itself (2004), data with reliability between 0.667 and 0.8 should only be used for "drawing tentative conclusions" (Krippendorff, 2004: 241).

- This is something that I already commented on above, but I think it is worth repeating. How did the authors come up with the typologies "survival," "self-expression," and "boundary"? Two of these come from the literature on post-modernity and survival vs. self-expression values by Inglehart and collaborators. However, why were these used as alternatives for "boundary"? Was it an arbitrary decision? Was it a result of prior analyses of the data? Also, if, for instance, WW2 can be considered both "survival" and "boundary," as the authors state, how do we distinguish between both? Do the authors even do this? An example would be the DK case in Table 1. WW2 is in the "boundary" cell but not the "survival" one. Whereas, for the UK case, WW2 is in both. How do the authors make this distinction or "choose" to which cell it belongs?

- Finally, one minor comment: I suggest using the median values for categoric variables in the online appendix C, the descriptive statistics per country. Regarding gender, I suggest the proportion of female or male respondents. For this type of categorical variable, mean values provide little information about the socio-demographics.

6 – Results

- I suggest avoiding linking "future events [one] wishes to see" to "future aspirations," as aspirations are far more complex, and I do not believe they can be measured by the question the authors use.

- Pg. 19, 2nd paragraph: "Clearly, these are different stories that represent different perceptions of the nation (…)." This is very confusing since the authors do not explain how they deal with the overlaps of themes, as I pointed out above.

- Pg. 19, 2nd paragraph: "Figure 1 shows a close proximity between the vote for a populist party and the boundary story across all four countries, indicating a correlation between holding this type of story and voting for a populist party."

1) Is the difference between the distances of respondents with boundary stories and the vote for other parties significant? The closest distance is between them and populist parties, as the graphs indicate, but is the difference between them and the other parties also significant?

2) I am far from an expert in MCA, but is this correlation significant?

- Pg. 19, 2nd paragraph: "It seems that the Survival story is less politicized, or less pertinent to political conflict, compared with the other two." I would suggest rephrasing this phrase, as the authors cannot infer that from their data (if survival stories are more or less politicized than the others).

- Pg. 19-20: "In all countries, the past and future story components cited by populist voters differ from those of mainstream voters, such that Immigration, Law, International Relations, Religion and Independence and the like are located closer to the populist vote, while Peace, Health, Rights, Sport, Education, and Science – further from populist and close to mainstream parties by a large margin." In at least 2 out of the four graphs, this is not the case, as some of the themes related to populism are closer to mainstream parties than populist ones.

- Pg. 24, 1st paragraph: for the same reasons I highlighted above, I do not think the authors have a moderating effect but rather a mediation effect.

- If possible, I would like to see in their robustness test the multinomial regression models with "Mainstream Left" as the category of reference. Since the authors are interested in the vote for Populist Radical Right Parties, having the Mainstream Left as the reference would make more sense. The results will not change, but the analysis would make more sense.

- Why is there no "populist attitudes" variable for the Netherlands? In Figure 2, the UK, DK, and the US have "populist attitudes" in their models, but the Netherlands does not. Why is that?

- I suggest the inclusion of "ideological extremism" as one of the covariates in their models. Ideological extremism has both an empirical and theoretical relationship with voting for the Far-Right (be it Radical or Extreme right). In this sense, I recommend the following papers for the operationalization of ideological extremism: Torcal & Magalhaes (2022).

- Page 26: I would be cautious in stating that "it seems that not only are national stories predictive of electoral behavior (even when controlling for populist attitudes and other variables that are known to explain voting for PPRPs) (…)" because populist attitudes are confounders and might be highly correlated with boundary national stories. If that is the case, although the authors' interpretation seems to be in the correct direction, the actual relationship and its interpretation are less straightforward and, therefore, need more caution.

- Page 26: "But it is a specific type of story that correlates positively with the vote for populist causes – one pivoting on in-group favoritism and the out-group hatred, the "us versus them" stance, and demarcated boundaries between the community and other groups, inside and outside the nation." The authors' description of what were considered "boundary national stories" as well as their method (questions regarding "past events" and "future events one wishes to see") does not allow them to infer that these events are connected to this type of narrative. From the authors' description and explanation, I do not see how events such as "WW2" or "End of Terror" might be related to "in-group favoritism and out-group hatred." It lacks a proper connection. The way it stands right now, I do not think the authors can affirm that.

- As mentioned before, I think the authors' models have a degree of multicollinearity that cannot be ignored. Although, nowadays, multicollinearity is not seen as a major problem anymore, certain cases need to be at least acknowledged. They hypothesize that "marginalized groups are more prone to translate the exclusionary boundary story into voting decisions, while its effect on the vote of their better of fellow citizens is less potent" (p. 26). Considering the literature, it seems like "marginalized groups" (and its indicators) might be highly correlated with Boundary National Story. Although I do not think this poses a problem to their paper, it is a relationship that should be noted.

- Page 26: The authors use three different variables to define "marginalized groups" (even if they are not creating typologies/categories from these variables). These are education, social class, and urban/rural divide.

1)	This applies to their initial models as well. The variable "social class" might be highly problematic. First, this variable could present a social desirability bias that could lead respondents to a more favorable self-presentation. I mean that respondents, to present themselves more positively, may respond in ways that make their "social class" look more positive to avoid embarrassment. Second, respondents might not properly distinguish between "upper" and "lower" middle class (for instance).

2)	Also, the urban/rural divide only makes sense in societies where it is salient. In this sense, the paper would benefit from a brief note on how salient or important this divide is for the national politics analyzed.

- Finally, the results presented in Figure 4 could be further improved with a more detailed analysis. Are the bars below each graph, histograms? If so, they seem to point toward a lack of common support in at least one of the cases. Not only that, but the interactions for DK seem to be non-linear. Therefore, I would suggest the authors take a closer look at their models and even test some alternatives to account for this non-linearity.

A good reference for that would be Hainmueller, Mummolo, and Xu (2021).

7 – conclusion

"this is the first study to probe the relationship between voters' narrative perceptions of their countries and support for extremist causes."

While PRRPs are indeed related to "extremist causes," there are two issues here:

1)	Vote for PRRPs should not be equated to "support for extremist causes." First, it would be wrong to treat voting for PRRP as supporting extremist causes, and the other, because the paper does not measure support for any specific cause.

2)	While Radical Right Parties can indeed be "extremists," there is a distinction between Radical and Extreme Right Parties. I suggest Cas Mudde's book on the Far Right for this distinction.

References (order that they appear):

Danieli, Oren, Gidron Noam, Shinnosuke Kikuchi & Ro’ee Levy. (2022). “Decomposing the Rise of The Populist Radical Right.” SSRN.

Mudde, Cas. (2019). The Far Right Today. Polity Press.

Berman, Sheri. (2021). “The Causes of Populism in the West.” *Annual Review of Political Science*.

Rovira Kaltwasser, Cristobal, and Steven Van Hauwaert. (2019). “The Populist Citizen: Empirical Evidence from Europe and Latin America.” EPSR.

Rico, Guillem, Marc Guinjoan, and Eva Anduiza. (2017). “The Emotional Underpinnings of Populism: How Anger and Fear Affect Populist Attitudes.” Swiss Political Science Review.

Hawkins, Kirk A. (2009). “Is Chávez Populist? Measuring Populist Discourse in Comparative Perspective.” Comparative Political Studies. Vol. 42(8).

Hawkins, Kirk A. (2010). Venezuela’s Chavismo and Populism in Comparative Perspective. Cambridge University Press. New York.

Poblete, Mario E. (2015). “Review Article: How to Assess Populist Discourse Through Three Current Approaches.” Journal of Political Ideologies.

Castanho Silva, Bruno. (2018). “Populist Success. A qualitative comparative analysis” In: Kirk A. Hawkins, Ryan E. Carlin, Levente Littvay, and Cristobal Rovira Kaltwassser (Eds.), *The Ideational Approach to Populism*.

Krippendorff, Klaus. (2004). Content Analysis: An Introduction to Its Methodology. Thousand Oaks: SAGE.

Torcal, Mariano and Pedro C. Magalhaes. (2022). “Ideological Extremism, perceived party system polarization, and support for democracy.” European Political Science Review.

Hainmueller, Jens, Jonathan Mummolo, and Yiqing Xu. (2021). “How Much Should We Trust Estimates from Multiplicative Interactive Models? Simple Tools to Improve Empirical Practice.” Political Analysis.
