# Supplementary material for: Voices from the margins: How national stories are linked with support for populist radical right parties
Source: PLoS One. 2024 Aug 12;19(8):e0305554. doi: 10.1371/journal.pone.0305554 (PMC11318891; doi:10.1371/journal.pone.0305554)
Supplement: Attachment — Submitted filename: Response to Reviewers_round2_plos1.docx [file pone.0305554.s005.docx]

Dear PLOS ONE Reviewers and Editorial Team,

Thank you for the invitation for a second round to revise and resubmit our paper, “Voices from the margins: How national stories are linked with support for populist radical right parties” (Ms. No. PONE-D-23-23023). We thank the reviewers for their feedback and specifically thank reviewer 1 and the editor for enabling us to work on a second round of revisions, as our previous handling of the comments was incomplete. In this version, we have shortened the introduction section for improved readability, conducted additional robustness checks, incorporated new literature from the social psychology field, and provided clearer explanations regarding the link between national stories and right-wing populism. Below we present the reviewers’ comments, followed by a discussion of how we address them in the revised manuscript. We apologize for the length of the memo, but we felt it was necessary in order to properly address all comments and suggestions. We will begin with addressing Reviewer 2's minor comments and then address the comments made by Reviewer 1.

*Response to Reviewer 2*

2.1 The reviewer notes that they “congratulate the authors for putting enormous effort into replying to my comments and suggestions and revising the manuscript accordingly. I think the manuscript is ready for publication”. They have one minor suggestion regarding the presentation of Figure 2, recommending consistency in the labeling of the graph, particularly using "Boundary" instead of "us/them".

**Response**: Point well taken. We fixed these inconsistencies in Figure 2. The renewed graph now uses the Boundary terminology, instead of us/them.

*Response to Reviewer 1*

We are grateful for your thoughtful engagement with our research and hope you will now find the paper appropriate for publication in PLOS ONE.

Reviewer 1 notes that they “appreciate the effort and the changes made by the authors.” They added that they “liked how the authors handled some of the comments regarding their research question, and I found that the changes improved heavily on the original manuscript.” However, they point out that the third and fourth points remain problematic, and many (if not all) minor comments were left unaddressed.

We appreciate the valuable feedback provided by the reviewer and apologize for overlooking the minor comments, which were included in an attached document that we inadvertently did not open. We are grateful for the opportunity for a second round of revisions. Below, we address each comment and provide our responses accordingly.

1.1 The reviewer expresses skepticism regarding the use of questions about past and future events as proxies for national stories. Specifically, the reviewer argues that "boundary national stories," primarily measure nationalism rather than populism as it emphasizes the division between the national community (in-group) and everyone outside it (out-group), without considering the distinction between elites and the people. Consequently, the reviewer suggests that any relationship between "boundary national stories" and support for Populist Radical Right Parties (PRRPs) would likely be driven by nationalism rather than populism.

**Response**: We appreciate the reviewer's engagement with our research design and have taken their feedback into account in the revised manuscript. Firstly, we clarify that the national stories in our study, captured through past and future events respondents deem significant, primarily relate to nationalism rather than populist narratives. This distinction is now emphasized in the manuscript to provide clarity. Our aim is to analyze respondents' perceptions of their nation and the relationship of these perceptions with voting behavior, particularly in relation to support for populist radical right parties (PRRPs).

Secondly, we acknowledge the role of nativism in the populist ideology, a point raised in the previous round of review, and we now explicitly state in the text that the association between national stories and voting for PRRPs is driven, for the most part, by nativism rather than populism (see pages 3,6,11). We also suggest avenues for future research to explore populist narratives by aligning elements of populism, such as people centrism and anti-elitism, with the components of people's narratives (see page 31).

Furthermore, we acknowledge that the operational definition of national stories may not fully capture the breadth of national narratives and encourage future research to enrich the operationalization by incorporating additional elements such as peoples’ perception of the most important national character. This broader approach could lead to a deeper understanding of the narratives shaping political behavior (see page 31).

1.2 The reviewer questions the relevance of using historical events like the Napoleonic Wars and the Industrial Revolution as proxies for "survival stories" in the context of assessing contemporary sentiments related to economic and physical security. They express doubt about whether these events still evoke such sentiments given the considerable time elapsed since their occurrence.

**Response**: We appreciate the reviewer's in-depth consideration of the national story construct and its definition. In response to this comment, we have provided further elaboration on our classification logic in the revised manuscript (see pages 17-19). The classification of past and future events (story components) like the "Napoleonic Wars" and the "Industrial Revolution" as belonging to the "Survival" category is based on their inclusion of economic or conflict-related elements.

It is important to note that our classification pertains to the stories themselves, not the individuals holding them. We use a ‘text-oriented’ approach to reading the text rather than ‘author-oriented’ since we cannot determine the individual intentions of every respondent. While such reading of the text has its pros and cons, it makes sense to assume that if a person orients her national stories around survival-related content (e.g. economic or conflict-related elements), even historical ones, it suggests a priority or at least an attention to survival framing, in comparison with respondents who chose events relating to, say, women's rights. Additionally, we should keep in mind that past events are but one component of our proxy for national story, and a "full" survival story must also involve an event related to survival in the future.

We believe that future research could advance the understanding, definition, and measurement of national stories. Despite this limitation, our study aligns with previous empirical endeavors aimed at assessing people's national stories through surveys, which is made more explicit in this version. We acknowledge the trade-off inherent in our approach: while we benefit from a broad dataset encompassing a representative sample of respondents across different countries, our main independent variable, the national story construct, is defined and measured thinly. Our operational definition, using a sequence of past and future events to represent the national story, is but one approach, and we anticipate that future studies will refine and expand upon this strategy (see our discussion section). Nonetheless, our research stands as the first, to our knowledge, to establish a link between national stories and the vote for PRRPs, and we hope to see further exploration in this area.

1.3 The reviewer wonders how “world war 2” marks the separation from other nations.

**Response**: Following this comment our revised manuscript underscores that conflicts and wars inherently establish boundaries between at least two opposing groups. Therefore, story components containing WW2 as a past event include boundary elements of "us versus them."

1.4 The reviewer asks whether the terms “survival” and “self-expression” come from studies in political culture and if so, they suggest acknowledging Inglehart's work in the text.

**Response**: The reviewer is correct. We rely on Inglehart and colleagues' work, and specifically write the following:

These three categories mirror broad political divisions in advanced democracies. The first two are based on the work of Inglehart and Welzel [87, 88] and of Norris and Inglehart [7] in the World Value Survey, in which people’s value priorities are aggregated at the country level, and according to these aggregations, each country is then placed on a two-dimensional value continuum. On the first axis, self-expression values are at one pole and survival values at the other. Self-expression values emphasize autonomy, human and minority rights, freedom, environmental protection, and quality of life, whereas survival values center around economic and physical security [88-90].

1.5 The reviewer asks that we better define populism and radical right. They believe that further clarification is needed beyond the added phrases in the introduction and section 3.

**Response**: Following the reviewer comment the revised manuscript includes a definition of populist radical right parties, and populism. We specifically state that, similar to other (and many) empirical studies, we rely on the ideational approach to populism, that sees populism as thin-centered ideology that its core features include: anti-pluralism, anti-elitism and the juxtaposition of a virtuous people against elites. On page 15 we write the following:

Relying on the ideational approach to populism that understands politics as a Manichean struggle between the will of the homogenous people and the corrupt elite and which argues that political sovereignty should reside with the ordinary people [4, 18], we utilize the PopuList data to classify parties as populist and radical right parties

Importantly, our definition is consistent with that of the PopuList data. This change complements previous revisions we have implemented. In the introduction, we now state:

This group of parties adhere to an ideology characterized by authoritarianism, nativism, and populism [4] and take radical positions on cultural issues with nativism, a combination of nationalism and xenophobia, being central to their ideology. They are regarded as populist, as they profess to represent the will of the people, as opposed to that of a putative “other,” commonly depicted as a corrupt and self-serving elite [5].

1.6 The reviewer suggests that the connection between "national stories" and "(radical right) populism" needs further development. They recommend establishing a clearer connection between these concepts and explaining how national stories are expected to relate to radical right populism.

**Response**: We appreciate the reviewer's call for clarification of our theoretical premises and value the opportunity to enhance our text accordingly. In the updated version of the paper, we provide a clearer explanation of the connection between Boundary stories and the support for populist radical right. We specifically write the following:

Our first theoretical expectation emanates from previous works on the importance of narratives, and specifically, on the idea that national stories reflect individuals’ political identities. If so, we expect to find differences between stories embraced by voters for mainstream and populist parties. Moreover, the demand side literature describes voters for PRRPs as having reverted to nativist values of exclusion and discrimination, in opposition to elites’ global and universalistic values [71], and as tending to glorify the past [66]. While populism and exclusionary nationalism, embodied in the Boundary type of national story, are analytically distinct constructs, they both play roles in the populist radical right. Populism revolves around the 'people as underdog' dynamic along an up-down axis, while exclusionary nationalism focuses on the 'people as nation' along an in-out axis. Despite their differences, both are integral components of the populist radical right. Previous works claim that right-wing populist parties mobilize potential voters by fueling feelings of resentment and anger, often leveraging "us-versus-them" dichotomies, whether between "the people" and the "corrupt elite" or through negative sentiments toward out-groups [72-74].

1.7 The reviewer advises caution regarding a statement suggesting that populist actors tend to perceive the world in terms of "us-versus-them" dichotomies, cautioning that this could be misleading given that populism requires anti-elitism.

**Response**: We thank the reviewer for this comment. We reworded this sentence, and now point to the strategy of PRRPs that often mobilize voters pitting them against the elites, or some others. We write the following:

Previous works claim that right-wing populist parties mobilize potential voters by fueling feelings of resentment and anger, often leveraging "us-versus-them" dichotomies, whether between "the people" and the "corrupt elite" or through negative sentiments toward out-groups.

1.8 The reviewer suggests splitting the paragraph on page 11 into smaller parts.

**Response**: Point well taken. We splitted the paragraphs into smaller parts.

1.9 The reviewer questions whether the classifications of stories as "survival," "self-expression," and "boundary" are mutually exclusive and whether the "boundary" type could also occur through values and norms. They inquire whether this would still fit within the "boundary" category or if it represents a different type of narrative.

**Response**: The reviewer's observation about our classification of story components into survival, self-expression, and boundary types is valid. These categories are not mutually exclusive; a story event can be classified under multiple types. For instance, World War II, a common past event, is classified as both survival and boundary. Importantly though, not all survival stories are boundary, and vice versa. This distinction is evident in our regression results, where survival tends to predict voting for mainstream parties, while boundary predicts voting for PRRPs.

Our classification is just one step in constructing the overall story. The second step involves combining past and future story components into a cohesive narrative that each individual holds. Respondents receive scores on the three indices of story types based on their composite narratives. For example, a respondent with a World War II past event and a peace future event would score 1 on all three indices, indicating elements of survival, boundary, and self-expression. Another respondent with a World War II past event and an economic prosperity future event would score 2 on survival, 1 on boundary, and 0 on self-expression.

1.10 The reviewer questions how story types such as "survival" and "self-expression" can encompass both material or economic concerns and the norms, goals, and values of a national group.

**Response**: Consider a respondent who regards the "Napoleonic Wars" as the most significant past event and the "end of terror" as a significant future event. These events are classified as belonging to the survival category, placing the individual at the highest level on the survival scale. For us, this choice of events and not others, signifies that the respondent prioritizes the survival (security) of the nation over other goals, norms and values.

1.11 The reviewer suggests that we consider stories as moderators or mediators in our models, depending on our theories/point of view.

**Response**: We thank the reviewer for their engagement with our argument and theoretical understanding on how stories may correlate with voter choice. We also truly think that more theoretical work is needed to connect stories with vote. In our manuscript, which to the best of our knowledge is the first to link stories with the vote for PRRPs, we link the two, with the moderating effect of voters’ marginal poisoning in society (low education, low status and the urban/rural divide), and show that boundary stories are indicative of the vote for PRRPs, only for those marginal groups in society. Those better off citizens who hold boundary stories, are less likely to vote for PRRPs. Following the reviewer comment we revised H2 (the hypothesis on the interaction effect) and accompanying text accordingly. We now write:

We argue, therefore, that to explain populist voting, it is necessary to take count of an interaction effect between the “us versus them” Boundary story and socio-demographic attributes such as education, social status and geographic periphery. Those who have lost out to globalization and whose socio-economic resources are limited will be more willing to translate their Boundary story into vote choice than their counterparts with better resources and socio-demographic positioning. Hence, our second hypothesis:

H2: The positive correlation between the Boundary story and support for PRRPs will be stronger within marginalized communities compared to their more privileged counterparts.

This hypothesis aligns with the economic grievances literature, arguing that socio-demographic attributes are important for populist support, but crucially adds an identity or cultural aspect – the type of national story one holds. We hypothesize that it is the combination of holding a Boundary story with belonging to a socially marginalized group that is positively associated with the vote for PRRPs. In the next section we empirically examine these hypotheses.

1.12 The reviewer suggests removing the variable "populist attitudes" from the models due to concerns about its measurement and construct validity. Specifically, they note that "populist attitudes would be a confounder in the relationship between boundary national stories and the vote for PRRPs". They also note that while some items may align with populist attitudes, others measure different concepts such as political efficacy.

**Response**: We thank the reviewer for this important comment. Our analysis reported in the main text is now performed without controlling for the "populist attitudes" scale. We present the analysis controlling that scale only in Appendix F. Following the reviewer's suggestion, we factor analyze all the items comprising that scale and report on the items comprising it (the factor analysis procedure generated one factor with eigenvalue greater than one, indicating that treating all items as a single scale or index would be appropriate) in Appendix D. Specifically, we write:

Populist attitudes. To what extent do you agree or disagree with the following statements? (Items presented in random order; for all items, 1=Strongly disagree and 5=Strongly agree; Items 5-8 were reverse-coded; scale reliability is between .64 and .76, depending on the country.) (1) Political parties are interested in my vote, not in my opinion; (2) Politicians do not understand what is going on in society; (3) There is a big gap between citizens and politics; (4) It doesn't matter whom you vote for, the situation remains the same; (5) Election campaigns give me enough information to make a choice; (6) The political candidates debated in an open and sincere campaign; (7) Parties can make a difference; (8) My friends and I have an influence on government policy.

**1.11** The reviewer suggests shortening the introduction to improve the flow of the paper

**Response**: We agree with this point and revised the introduction section accordingly. This involved trimming certain parts, reframing others, and relocating some content to more suitable sections.

**1.12** The reviewer questions the adequacy of observational data to measure the extent to which respondents are affected by boundary stories, as implied by research question 2.

**Response**: We thank the reviewer for this comment. Following it, throughout the manuscript, we now acknowledge the limitations of inferring causality from observational data. Also, in the conclusion, we emphasize the need for future experimental studies to address this limitation.

1.13 The reviewer suggests clarifying the terminology used to describe individuals at the bottom of society's power structure. They note a transition from "powerless" in the introduction to "marginalized" later on and suggests that we pick and choose one of these terms.

**Response**: In response to this feedback, we have revised the text to use "marginality" instead of "powerless." This decision aligns with the literature on the emergence and support for populist radical right parties, which commonly employs the term "social marginalization." The theories of relative deprivation and modernization losers highlight the significance of social marginalization in understanding the dynamics of radical right-wing populist parties. We further explain the term marginality and how it is used in the paper. We write the following:

Some studies define marginalization in objective terms, as belongingness to a group that is deprived of certain resources [76-78, 48], while others define it as the subjective sense of low societal status or lack of recognition [79-81, 11]. Yet, while these studies delve into different facets of "left behindness," they do not directly pinpoint the primary motivator behind support for PRRPs. We propose that socially marginalized individuals, who recognize themselves as belonging to low-status groups, are often driven to exclude others to enhance their own sense of belonging within a community [82]. In other words, marginalized people might cope with their subordinate position and at the same time strive to gain greater acceptance to mainstream society by distancing themselves, physically or psychologically, from other marginalized groups, and labeling the latter as outsiders and a threat to the community

1.14 The reviewer suggests that we add references to our claim that our results “challenge early studies that inferred electoral motives of voters in Western Democratic societies directly from their material life conditions."

**Response**: We thank the reviewer for their comment and added references to substantiate our claim.

1.15 The reviewer points out that Coffé's study focuses on Radical Right Parties (RRPs), not specifically PRRPs and that while there is overlap between RRPs and PRRPs, it's important to distinguish between them. They suggest that if we cite Coffé's work to describe the profile of PRRP supporters, it should be explicitly acknowledged and clarified.

**Response**: We acknowledge the reviewer's point and have replaced the citation of Coffé's work on the radical right with Oesch's research on the support for PRRPs among the working class. We also note that it is not only their position at the labor market, but a bigger argument of vulnerability of manual and routine workers to the penetration of immigrants, automation, and the offshoring of jobs.

1.16 The reviewer suggests using a different reference than Becker et al. (2017) for the claim that PRRPs supporters predominantly have "low-to-intermediate levels of education."

**Response**: We replaced Becker citation with Danieli et al 2022, as suggested by the reviewer.

1.17 The reviewer notes that the introduction's focus on socio-structural attributes of PRRP supporters misses the broader context. They argue that these attributes are often consequences of deeper feelings of insecurity and economic grievances, which can lead to nativist sentiments and support for Populist Radical Right Parties. The reviewer recommends acknowledging this broader perspective to enhance the manuscript.

**Response**: We thank the reviewer for encouraging us to expand the discussion on the economic explanations for the support bases of PRRPs. We added additional text highlighting how socio-structural attributes of marginality can lead to insecurity and vulnerability in the labor market which may fuel anti-immigrant feelings and contribute to the support in PRRPs. We wrote the following:

According to some within this strand of research, anti-immigrant sentiments promoted by PRRPs are framed to appeal to those who have lost out due to globalization, usually blue-collar male workers whose jobs have been put at risk by the influx of manual immigrant workers [28, 29]. In the US, low-skilled workers were found to support restrictions on immigration more than their high-skill counterparts [30]. In the European context, studies show that individuals employed in shrinking sectors are more likely to oppose immigration than those employed in growing sectors [31], and relatedly, occupations with few exit options and low skill transferability are more sensitive to potential competition with migrants [32]. In summary, the economic grievances approach highlights how social marginalization in a globalized world can amplify insecurity and vulnerability in the labor market. Globalization and modernization increase the risk of job loss, lower-paying employment, or replacement by immigrants, particularly for manual workers and those with lower education levels. Consequently, economic grievances and insecurity often fuel anti-immigrant nativism, contributing to support for PRRPs.

1.18 The reviewer challenges our assertion that PRRPs lack a coherent economic ideology, arguing that they do present identifiable economic ideologies within the broader right-wing and far-right party families.

**Response**: We thank the reviewer for this comment. Consequently, we now highlight in the revised text that an important part of the history of a lot of populist radical-right parties is that they shifted from the so-called “winning formula” (combining economically right-wing positions with cultural-conservative positions) to more centrist economic positions aimed at attracting broader support. We back this assertion by citing Kitschelt and Mcgann (1997) and de Lange (2007). In the US, the Trump presidency presented a unique case where liberal and conservative issue positions were presented and backed by the president almost simultaneously. We cite Barber and Pope (2019) to back this assertion.

1.19 The reviewer recommends reformulating the sentence on page 5 where we claim to empirically demonstrate how grievances are channeled into populist agendas

**Response**: We reworded this sentence and now provide a more accurate description of our study.

1.20 The reviewer states that the draft is based on concepts such as "populism" and "radical right," since it focuses on "populist radical right parties." However, it does not define either of these concepts.

**Response**: We have dealt with this comment on the first round of revisions, but understood that the reviewer wants to hear more on the definition and approach to populism and to radical right populist parties that we take. Here is what we did in the first and the second rounds of revisions:

We now include a definition of radical right populist parties in the introduction:

This group of parties adhere to an ideology characterized by authoritarianism, nativism, and populism [4] and take radical positions on cultural issues with nativism, a combination of nationalism and xenophobia, being central to their ideology. They are regarded as populist, as they profess to represent the will of the people, as opposed to that of a putative “other,” commonly depicted as a corrupt and self-serving elite [5].

Moreover, in Section 3 ("National stories and politics") we added a paragraph on nativism, a key feature in the ideology of populist radical right parties.

A key feature of the PRRP ideology is nativism, a belief that "states should be inhabited exclusively by members of the native group (“the nation”) and that nonnative (or “alien”) elements, whether persons or ideas, are fundamentally threatening to the "homogeneous nation state" [75]. Indeed, the distinction between “us” and “them” is at the ideological core of PRRPs and is touted by populist leaders, who shift all blame from “the innocent people” to either out-groups or “corrupt elites.”

Lastly, in the “data and measurement” section we now write the following:

Relying on the ideational approach to populism that understands politics as a Manichean struggle between the will of the homogenous people and the corrupt elite and which argues that political sovereignty should reside with the ordinary people [4, 18], we utilize the PopuList data to classify parties as populist and radical right parties.

1.21 The reviewer asks that we rephrase our sentence that "scholars have not been able to establish empirically consistent connections between individuals' economic circumstances such as income, wealth, or employment status and the propensity to support populism" and that our results corroborate these outcomes, to make our point clearer.

**Response**: We thank the reviewer for pointing this out and consequently rephrased our sentence to make clear that our results corroborate the lack of consensus in the literature.

1.22 The reviewer notes that the sentence:"Second, it has been established that people tend to identify more with high- than low-status groups. Thus, support for populist causes cannot be fully, nor indeed directly, accounted for by socio-demographic marginality." is hard to follow.

**Response**: We revised this sentence.

1.23 The reviewer suggests that we cite Rico et al. (2017) in the discussion on the cultural and economic explanations to populism.

**Response**: We thank the reviewer for this comment. The revised manuscript now cites this work in the discussion of the two approaches. We write:

Rico et al. (2017) [46] show how individual levels of populist attitudes are related to emotional reactions to the economic crisis in Spain and that populist attitudes are influenced by feelings of anger rather than anxiety.

1.24 The reviewer recommends linking the studies summarized in section 2 to populism by highlighting how declining trust in political institutions can foster support for populism.

**Response**: We completely agree with the reviewer's suggestion and consequently wrote the following sentence that summarize the paragraph’s narrative:

The above studies establish a more complexed link between economic attributes and populism, one that is mediated by negative emotions, identities or a declining trust in political institutions.

1.25 The reviewer suggests that we rephrase the sentences at the end of section 2 (“... this paper aims to identify the various characteristics and factors that render one's vote susceptible to such narratives, along with the features of a national story that increase the likelihood of populist leanings.”).

**Response**: Following previous comments by the reviewer, we rewrote section 2 entirely. The above quoted sentence was deleted from the revised version.

1.26 The reviewer suggests revising the title of section 3 and removing "political behavior" as it does not focus significantly on actual political behavior.

**Response**: We understand the reviewer's comment, and changed the title of section 3 to “national stories and politics”, as this section reviews recent literature that links between the two.

1.27 The reviewer questions our claim that populist ideas are anchored in national identity, suggesting that while national identity may contribute to populism by uniting the "us" as "the people," it's not necessarily the sole anchor for populist ideas.

**Response**: We agree with the reviewer that we were not precise in that assertion in the previous draft of the manuscript. We now emphasize the connection between national identity and nativism, which is a component of right-wing populism. We specifically write the following:

Moreover, the demand side literature describes voters for PRRPs as having reverted to nativist values of exclusion and discrimination, in opposition to elites’ global and universalistic values [71], and as tending to glorify the past [66]. While populism and exclusionary nationalism, embodied in the Boundary type of national story, are analytically distinct constructs, they both play roles in the populist radical right. Populism revolves around the 'people as underdog' dynamic along an up-down axis, while exclusionary nationalism focuses on the 'people as nation' along an in-out axis. Despite their differences, both are integral components of the populist radical right. Previous works claim that right-wing populist parties mobilize potential voters by fueling feelings of resentment and anger, often leveraging "us-versus-them" dichotomies, whether between "the people" and the "corrupt elite" or through negative sentiments toward out-groups [72-74].

1.28 The reviewer suggests adding a reference to support the claim made in the second paragraph of page 10 regarding populist parties mobilizing potential voters by fueling feelings of resentment and anger.

**Response**: We added references supporting our assertion.

1.29 The reviewer asks that we explain/define what the boundary story is.

**Response**: Following this and other related comments, the revised manuscript now defines throughout the text that leads to the first hypothesis what boundary story is. In the abstract we write that: “This article explores the influence of "boundary national stories," emphasizing clear distinctions between "us" and "them" in national identity, on voting patterns”. In the introduction we add that: “the Boundary story defines the national community relationally and negatively, in terms of exclusion.” and that “individuals who embrace national stories in the Boundary category, emphasizing conflict between a political community and other groups, are more likely to vote for PRRPs”.

1.30 The reviewer argues that no references are given in the text to how the cultural explanations fit our research/would be used to answer our research questions.

**Response**: The revised manuscript now cites Norris & Inglehart's "cultural backlash" and Bartles "Democracy Erodes from the Top" book.

As marrying cultural and economic grievances are at the heart of our endeavor in the paper, we apologize for the oversight in not making this clearer. In the revised manuscript, we explicitly connect cultural and economic grievances. In the introduction, we highlight that the national stories capture a cultural, identity-based element, while economic grievances are reflected in individuals' attributes such as social status, education, and urban/rural divide. We write:

This paper argues that the explanation for the emergence and success of right-wing populism lies not only in the cultural factors or the economic ones, but at the intersection of both, with the perception of the nation serving as an important cultural factor.

And we continue to explain what we mean by “the perception of the nation”

We suggest that a good predictor of people’s vote choice is their perception of their nation, as indicated by the types of stories they tell about their political community, its origin, history, and anticipated future prospects….

And again, in the conclusion we write:

The literature on the demand side of populism has grappled with the question regarding the relative role of economic and cultural factors, and how they might be linked. Our study feeds into this fundamental debate by exploring the link between voters’ socio-demographic attributes and their national identity couched in the national stories they embrace

1.31 The reviewer asks us to define what constitutes "low status" more explicitly, particularly whether it refers to economic terms or other dimensions. Additionally, they suggest that we clarify what constitutes marginalized groups and how to define/measure it.

**Response**: We thank the reviewer for raising this point. In the revised manuscript, we have provided a clearer definition of marginalized groups, drawing from literature on the "left behinders" and social deprivation. We propose measuring marginalization through three proxies: education, geographic periphery, and social status. Our theoretical framework posits that individuals situated at the bottom of society's power structure, and thus socially marginalized, will be more responsive to the boundary story in their voting behavior. This clarification strengthens the linkage between marginalization and support for PRRPs. On page 13 we write the following:

We argue that a Boundary story is likely to be more central to the self-definition of individuals stationed along an in-group’s periphery, in terms of geography (far flung), education (medium and low) and status (low), and are in a sense marginalized. Some studies define marginalization in objective terms, as belongingness to a group that is deprived of certain resources [76-78, 48], while others define it as the subjective sense of low societal status or lack of recognition [79-81, 11]. Yet, while these studies delve into different facets of "left behindness," they do not directly pinpoint the primary motivator behind support for PRRPs. We propose that socially marginalized individuals, who recognize themselves as belonging to low-status groups, are often driven to exclude others to enhance their own sense of belonging within a community [82]. In other words, marginalized people might cope with their subordinate position and at the same time strive to gain greater acceptance to mainstream society by distancing themselves, physically or psychologically, from other marginalized groups, and labeling the latter as outsiders and a threat to the community.

1.32 The reviewer suggests that the paper would benefit from incorporating articles that adopt a discursive perspective on populism (e.g., Hawkins (2009, 2010) and/or Poblete (2015)), particularly those focusing on the success of populist parties.

**Response**: We thank the reviewer for suggesting these important contributions. The updated version of the paper cites Poblete’s work. We did not cite the papers by Hawkins on populism on the left as ours focuses on populism on the right.

1.33 The reviewer questions the relationship between national stories and "marginalized groups," suggesting a potential issue of multicollinearity. They propose that while boundary-national stories might affect the self-definition of marginalized groups, as we suggest, these groups might also express such stories because of their marginalized status.

**Response**: We appreciate the reviewer's insightful observation regarding the potential multicollinearity between the Boundary story and belonging to socially marginalized groups. In response, we conducted supplementary analyses to examine the distribution of the Boundary story among various demographic groups, including marginalized and better-off individuals categorized by education level, social status, and residential location (rural vs. urban).

Our findings reveal that while there may be a tendency for marginalized groups to align with the Boundary story, the degree of association between the two is only moderate at best, and often exhibits low correlation. Specifically, while it is true that marginalized groups may more frequently endorse the Boundary narrative compared to better-off groups, the relationship between the two is not overwhelmingly strong.

In our paper, we do not hypothesize about the prevalence of the boundary story among different groups, but rather assert that the interplay between individuals' societal positioning, such as belonging to marginalized groups, and their embrace of the Boundary story, significantly influences their propensity to support PRPPs. In other words, for us, it is not a matter of composition effect but rather an interaction effect. We argue that objective indicators of marginalization, including lower education levels, lower social status, and peripheral residency, combined with a national narrative characterized by an "us versus them" dynamic, contribute to increased support for the radical right.

Our supplementary figures below show that various demographic groups may also adopt narratives featuring Boundary characteristics. But, our argument focuses on the combination of marginalized status and adherence to such narratives that drives individuals toward populist radical right voting behavior. These findings underscore the nuanced relationship between societal positioning, narrative engagement, and political preferences, as elucidated in our paper.

1.34 The reviewer suggests that the hypothesis on page 13, concerning the interaction between socio-demographic attributes and the Boundary type of story in influencing populist support, might not reflect a moderation effect as claimed, but rather a mediation effect.

**Response**: We thank the reviewer for this comment. In the revised version we rephrased both the hypothesis (we now claim for a correlation and not causation) and the sentences following this hypothesis. We write:

We argue, therefore, that to explain populist voting, it is necessary to take count of an interaction effect between the “us versus them” Boundary story and socio-demographic attributes such as education, social status and geographic periphery. Those who have lost out to globalization and whose socio-economic resources are limited will be more willing to translate their Boundary story into vote choice than their counterparts with better resources and socio-demographic positioning. Hence, our second hypothesis:

H2: The positive correlation between the Boundary story and support for PRRPs will be stronger within marginalized communities compared to their more privileged counterparts.

This hypothesis aligns with the economic grievances literature, arguing that socio-demographic attributes are important for populist support, but crucially adds an identity or cultural aspect – the type of national story one holds. We hypothesize that it is the combination of holding a Boundary story with belonging to a socially marginalized group that is positively associated with the vote for PRRPs. In the next section we empirically examine these hypotheses.

1.35 The reviewer inquires about the case selection and the sampling method used for the online data collection.

**Response**: We have corrected this omission by adding a paragraph to the “Data and measurement" sub-section, explaining our case selection. We also added Appendix A where we elaborate on survey companies, sampling techniques, and descriptive statistics of the samples.

Specifically, we wrote:

The study tests the relationship between populist voting and national stories in four countries with different histories – notably, with respect to immigration and PRRP support: Denmark, the Netherlands, the United Kingdom and the United States. These countries were chosen also because they are all developed democracies, but differ in their political systems as presidential vs. parliamentary democracies, as well as their electoral systems and district magnitude (e.g., extreme proportionality in the Netherlands vs. single-member plurality in the UK and the US). The above variations across the countries sampled make it possible to extrapolate the results to other developed democracies and draw general inferences regarding the relationship between national stories and vote choice.

1.35 The reviewer notes that we mentioned in the text that we control for populist and nativist attitudes, and in another comment suggested dropping this control from the analysis.

**Response**: In response, we have removed the populist attitudes scale from the main analysis. However, we have included it as a control variable (and for robustness) in the Appendix, where Appendix D provides an explanation of how the scale was created.

1.36 The reviewer questions the validity of the party classifications used, which is based on the work of Laver, Gallagher, and Mair (2011) and Parlgov data (2016). They express concerns regarding the lack of clarity in Parlgov's criteria for classifying parties as "populist". The reviewer emphasizes the importance of transparency and reproducibility in addressing this issue. Additionally, they recommend considering alternative party classifications such as Poppa or the PopuList, as a robustness test, due to their clearer criteria and descriptions.

**Response**: Following the reviewer’s comment, we now rely on the PopuList data to categorize parties to PRRPs. The PopuList classification fits perfectly with our ideational approach to populism.

1.37 The reviewer notes that asking only one past event and one future event may not be the most reliable method to assess respondents' national stories. They express concern that external factors such as exposure and context could influence respondents' answers, potentially leading to responses that do not accurately reflect their true national story.

**Response**: The reviewer rightly points out a limitation in our methodology for gauging people's national stories: by aggregating their most significant past events and future aspirations for their nation, we provide a practical yet somewhat simplistic approach. It serves as a reduction or proxy for national stories, and future theoretical work should aim to develop more nuanced and precise measurement methods. We acknowledge that noise may indeed filter into our measurement strategy. Therefore, we have addressed these limitations in the discussion section and propose further research to delve deeper into this subject. Although a thin proxy, it is the first paper to show a link between people's perception of their nation and their voting behavior.

1.38 The reviewer queried about the rationale behind selecting the typologies of "survival," "self-expression," and "boundary". Additionally, they highlight the challenge of distinguishing between events categorized as both "survival" and "boundary," such as World War II, and seek clarification on how we make such distinctions.

**Response**: We thank the reviewer for this comment as it pushed us to be clearer on our typology and to revise Table 1 accordingly. We now write in the revised manuscript that the three-story types mirror broad political divisions in politics. As the reviewer alluded, the first two categories are drawn from research by Inglehart and Welzel as well as Norris and Inglehart – self-expression story types emphasize autonomy, human and minority rights, freedom, environmental protection, and quality of life, while survival story types prioritize economic and physical security. But to these two categories we add a third one, the Boundary story type, which, together with the self-expression story type, can be seen as mirroring another political division in advanced democracies — a division between universalism and particularism or between cosmopolitanism and communitarianism. Together the three categories represent tripolar political divisions in affluent societies - the economic and security dimension (Survival), the identity dimension (Self-expression) and the community nationalistic dimension (Boundary). We write the following:

These three categories mirror broad political divisions in advanced democracies. The first two are based on the work of Inglehart and Welzel [87, 88] and of Norris and Inglehart [7] in the World Value Survey, in which people’s value priorities are aggregated at the country level, and according to these aggregations, each country is then placed on a two-dimensional value continuum. On the first axis, self-expression values are at one pole and survival values at the other. Self-expression values emphasize autonomy, human and minority rights, freedom, environmental protection, and quality of life, whereas survival values center around economic and physical security [88-90]. The Boundary and Self-expression story types also tap important political divisions in affluent societies, usually termed as the universalism-particularism or cosmopolitanism-communitarianism divide [91]. Boundary national stories establish a division between "the people," or the national community – “us” – and everyone outside it – “them.” These stories inherently position other nations as outgroups, emphasizing a separation between them and the ingroup (the nation).

1.39 The reviewer recommends utilizing median values for categorical variables in the online Appendix, along with providing the proportion of female and male respondents.

**Response**: As suggested by the reviewer, we replaced the mean values for gender with the percentage of women in the sample, and now report the percentages of response categories for the variable of social class, education and rural/urban divide.

1.40 The reviewer advises against equating "future events one wishes to see" with "future aspirations," arguing that aspirations cannot be adequately captured by our question.

**Response**: Point well taken. We replaced "aspirations" with "future events one wishes to see".

1.41 The reviewer finds a statement on page 19, second paragraph ("Clearly, these are different stories that represent different perceptions of the nation (…)."), confusing because of overlapping themes in the analysis.

**Response**: Point well taken. We revised the sentence and included a footnote acknowledging that while there may be some overlap in classification, particularly regarding past events categorized under both Survival and Boundary, the distinct positions of the three-story types in the MCA figures underscores their differences and the different correlation of these story types with voter choice.

1.42 With regard to the MCA, the reviewer asks whether the difference between the distances respondents with Boundary stories and the vote for other parties is significant and whether the correlation between the Boundary story and vote for PRRPs is significant.

**Response**: We thank the reviewer for this inquiry. The MCA output does not provide statistical significance for the distances between response categories; it only presents the magnitude of the distance both mathematically and graphically. We chose to present these figures as suggestive evidence for a consistent (across all four cases) link between story types and voter choice. This link is further explored via regression analysis in the second empirical phase, where we asses a vote choice model (voting for PRRPs) as a function of Boundary and other story types, controlling for attitudinal and demographic variables known to correlate with the vote for the populist radical right.

1.43 The reviewer recommends rephrasing a statement from the second paragraph of page 19, emphasizing that we cannot conclusively infer from our data whether Survival stories are more or less politicized compared to the other story types.

**Response**: We agree and rephrased the sentence accordingly. We now write: “On the other hand, the Survival story, which centers mainly on economic and security issues, is located on the vertical axis, and explains less variance. This could suggest that the Survival story is less pertinent to partisan divisions, compared with the other two.”

1.44 The reviewer points out a discrepancy between the claim made on pages 19-20, stating that themes cited by populist voters are consistently closer to the populist vote compared to mainstream voters. They note that “In at least 2 out of the four graphs, this is not the case, as some of the themes related to populism are closer to mainstream parties than populist ones”.

**Response**: We appreciate the reviewer's observation. As a result, we have rectified the errors and revised the sentence accordingly.

1.45 The reviewer suggests changing the reference category of the multinomial regression models to "Mainstream Left" in the robustness test. They argue that using the Mainstream Left (instead of the Mainstream Right) as the reference category would be more appropriate and enhance the coherence of the analysis.

**Response**: We changed the reference category in these models (presented in Appendix E).

1.46 The reviewer questions the absence of a "populist attitudes" variable for the Netherlands in the analysis. They note that while the UK, Denmark, and the US include this variable in Figure 2, it is missing for the Netherlands.

**Response**: The survey for the NL did not include the battery of questions composing the populist attitudes scale. We nevertheless and following the suggestion of the reviewer omitted this scale from the main analysis. We did however include the populist attitudes scale in the robustness tests (Appendix E), and also elaborate (Appendix D) on how this scale was created and what are the items included in the battery.

1.47 The reviewer recommends incorporating "ideological extremism" as a covariate in the models, and suggests referring to Torcal & Magalhaes (2022) for guidance on operationalizing ideological extremism.

**Response**: We thank the reviewer for this suggestion. While we recognize the empirical and theoretical relevance of extremism, we emphasize that it is specifically right-wing ideological extremism that may correlate with the vote for PRRPs. Given that Torcal & Magalhaes (2022) conceptualize and measure extremism without specifying ideological direction (left or right), we chose to control for respondents' left-right self-placement in our models. Our results indeed indicate a positive correlation between left-right self-placement and the vote for PRRPs, with respondents leaning further to the right more likely to vote for PRRPs.

1.48 The reviewer advises caution regarding the assertion that national stories predict electoral behavior independently of populist attitudes and other variables. They highlight the potential for confounding, particularly if populist attitudes are highly correlated with boundary national stories.

**Response**: We thank the reviewer for pointing this out. Following another comment by the reviewer, we rerun our regression models omitting the populist attitudes scale. We revised the text accordingly.

1.49 The reviewer points out a potential issue of multicollinearity in our models, particularly concerning the relationship between marginalized groups and the boundary national story. While acknowledging that multicollinearity is less concerning nowadays, they suggest that this relationship should be acknowledged due to its potential significance.

**Response**: This point has been raised also elsewhere. Following this concern, we checked for multicollinearity between being marginalized (low education, low status and living in rural areas) and holding Boundary national story. The correlation between the two is at best moderate, and for the most cases is very low (<.2). We attached figures displaying the distribution of the Boundary story among the marginalized and better off citizens for all four countries.

1.50 The reviewer expresses concern regarding the variable "social class," suggesting it may pose several problems. First, they note the potential for social desirability bias, wherein respondents may provide more favorable self-presentations to avoid embarrassment. And second, they highlight the possibility that respondents might struggle to accurately distinguish between different social class categories, such as "upper" and "lower" middle class.

**Response**: We share the reviewer's concern about social desirability bias and consequently added a footnote (#9) noting this concern. However, we adhere to the approach followed by others, such as Gidron and Hall, in defining and measuring social class subjectively based on respondents' self-assessment. We believe it is reasonable to assume that if respondents misrepresent their social class, they are likely to only slightly upgrade their status, for example, from "skilled working class" to "lower middle class." Even in such cases, they would still be categorized within the lower bound of social class categories.

1.51 The reviewer suggests providing a brief note on the salience or importance of the urban/rural divide for the national politics analyzed, as this divide may only be relevant in societies where it is prominent.

**Response**: We thank the reviewer for this suggestion. We added a footnote noting that the urban/rural electoral divide is highly pronounced in the majoritarian democracies of the UK and North America, where the republican and conservative parties rely on support from rural areas, but it is also evident in Denmark and to a lesser extent in the Netherlands. We also cite a recent CPS paper by Huijsmans & Rodden 2024 to support our claim (see fn #10).

1.52 The reviewer suggests that the results presented in Figure 4 could be enhanced with a more detailed analysis. They recommend that we supply further information on the bars below each graph and check for nonlinearity.

**Response**: Point well taken. The revised manuscript now provides a full description of the graphs presented in Figure 4, including an explanation that the bars below the predicted margins are histograms displaying the distribution of the moderators. Additionally, we have rerun the figure, ensuring to check for linearity using the interflex package, as suggested by Hainmueller, Mummolo, and Xu (2021). Indeed, the case of Denmark shows a non-linear relationship, though this does not undermine the main effect of the interaction.
